# Impacts of Plu kaow (*Houttuynia cordata* Thunb.) Ethanolic Extract on Diabetes and Dyslipidemia in STZ Induced Diabetic Rats: Phytochemical Profiling, Cheminformatics Analyses, and Molecular Docking Studies

**DOI:** 10.3390/antiox13091064

**Published:** 2024-08-30

**Authors:** Shaikh Shahinur Rahman, Anuwatchakij Klamrak, Napapuch Nopkuesuk, Jaran Nabnueangsap, Piyapon Janpan, Kiattawee Choowongkomon, Jureerut Daduang, Sakda Daduang

**Affiliations:** 1 Division of Pharmacognosy and Toxicology, Faculty of Pharmaceutical Sciences, Khon Kaen University, Khon Kaen 40002, Thailand; shahin@anft.iu.ac.bd (S.S.R.); anuwat_kla@yahoo.com (A.K.); napapuch.aom25@gmail.com (N.N.); j.piyapon@kkumail.com (P.J.); 2 Department of Applied Nutrition and Food Technology, Faculty of Biological Sciences, Islamic University, Kushtia 7003, Bangladesh; 3 Salaya Central Instrument Facility RSPG, Research Management and Development Division, Office of the President, Mahidol University, Nakhon Pathom 73170, Thailand; jaran.nab@mahidol.edu; 4 Department of Biochemistry, Faculty of Science, Kasetsart University, Bangkok 10900, Thailand; fsciktc@ku.ac.th; 5 Department of Clinical Chemistry, Faculty of Associated Medical Sciences, Khon Kaen University, Khon Kaen 40002, Thailand; jurpoo@kku.ac.th; 6 Protein and Proteomics Research Center for Commercial and Industrial Purposes (ProCCI), Khon Kaen University, Khon Kaen 40002, Thailand

**Keywords:** Plu kaow, *Houttuynia cordata*, diabetes, hyperlipidemia, molecular docking, cheminformatics, phytochemical profiling

## Abstract

The increasing prevalence of diabetes and dyslipidemia poses significant health challenges, impacting millions of people globally and leading to high rates of illness and death. This study aimed to explore the potential antidiabetic and hypolipidemic effects of Plu kaow (*Houttuynia cordata* Thunb.) ethanolic extract (PK) in streptozotocin (STZ) induced diabetic rats, focusing on its molecular mechanisms. Diabetes was induced in fasting Long Evans rats using streptozotocin (65 mg/kg b. w.), with glibenclamide (5 mg/kg/day) used as the standard experimental drug. The treated groups received oral supplementation of PK (500 mg/kg/day) for 28 days. The study evaluated blood glucose levels, lipid status, body weight, liver, kidney, and heart function biomarkers, antioxidant activity, and histological examination of various organs. Additionally, untargeted metabolomics, cheminformatics, and molecular docking were employed to elucidate the probable mechanisms of action of PK. Based on metabolomic profiling data, the PK was found to contain various putative antidiabetic agents such as kaempferol 7-neohesperidoside, isochlorogenic acid C, rutin, datiscin, and diosmin and they have been proposed to significantly (*p* < 0.001) reduce blood glucose levels and modulated hyperlipidemia. PK also improved the tested liver, kidney, and heart function biomarkers and reversed damage to normal pancreatic, liver, kidney, and heart cells in histological analysis. In conclusion, PK shows promise as a potential treatment or management option for diabetes and hyperlipidemia, as well as their associated complications in diabetic rats.

## 1. Introduction

Diabetes mellitus (DM) is a group of disorders with persistent hyperglycemia that have multiple aetiologies [1]. It has emerged as a major health issue in recent years and ranked as the top cause of mortality throughout the world [2]. The International Diabetes Federation (IDF) reports that the worldwide number of diabetic persons is currently at 537 million, and it is projected to reach 783 million by 2045 [3]. This progressive disease receives high attention due to associated complications like atherosclerosis, stroke, and peripheral vascular disease [4].

Dyslipidemia is a prevalent condition in patients with type 2 diabetes, impacting approximately 72–85% of patients [5]. Diabetic dyslipidemia refers to a group of lipoprotein abnormalities characterized by hypertriglyceridemia (TG), and high levels of low-density lipoprotein (LDL), along with a reduction in high-density lipoprotein-cholesterol (HDL) levels [6]. The specific mechanism that causes lipoprotein abnormalities in diabetes is not yet fully understood. However, it is known that long-term hyperglycemia can damage the vascular endothelium. This damage reduces the activity of functional lipoprotein lipase, ultimately leading to higher levels of triglycerides (TG) and lower levels of high-density lipoprotein cholesterol (HDL-C) [7]. Another possible cause of lipoprotein dysfunction is the malfunctioning of pancreatic beta cells. This is often attributed to glucotoxicity and high levels of free fatty acids, which can also increase the oxidation of lipoproteins, leading to accelerated atherogenesis [8].

Insulin resistance is often linked with a significant rise in the levels of very low-density lipoproteins (VLDL) due to the reduced activity of lipoprotein lipases [9]. Moreover, a change occurs in the makeup of high-density lipoproteins (HDL), leading to a loss of their biological functions and transforming them into a dysfunctional lipoprotein [10]. In recent years, research has combined traditional plant-based medicines with modern scientific methods to develop drugs for treating various diseases, such as hyperglycemia, diabetic dyslipidemia, and oxidative damage in DM, which offer new possibilities with minimal or no side effects [11].

Plu kaow or *Houttuynia cordata* Thunb. is a perennial plant that belongs to the ‘Saururaceae’ family. It is widely found in East Asia and is commonly used as a vegetable in local cuisine. It has been used for centuries as a folk medicine and is also utilized in the preparation of fermented beverages, nutraceuticals, feed, and cosmetics [12]. It is rich in various nutrients such as protein, soluble sugar, fat, volatile oils, total flavonoids, and trace elements like sulfur (S), phosphorus (P), potassium (K), magnesium (Mg), calcium (Ca), iron (Fe), zinc (Zn), and copper (Cu) [13,14]. The inductively coupled plasma mass spectrometry (ICP-MS) method has been utilized in recent studies to detect heavy metals in plu kaow. These metals include manganese (Mn), zinc (Zn), copper (Cu), cobalt (Co), titanium (Ti), stannum (Sn), antimony (Sb), barium (Ba), chromium (Cr), nickel (Ni), arsenic (As), lead (Pb), mercury (Hg), and cadmium (Cd), with concentrations ranging from 0.15 to 695 g·L^−1^ [13,15].

To date, researchers have identified 603 phytoconstituents in plu kaow, categorized into 11 types of compounds, including aliphatic (259), terpenoids (158), flavonoids (2), aromatics (42), alkaloids (42), phenylpropanoids (20), amides (17), steroids (15), saccharides (8), glycosides, phenolic acids (8), and other categories (8) of compounds [16]. Flavonoids and volatile oils were found to have the highest pharmacological activities among the various constituents [17]. Moreover, researchers pointed out that α-pinene, β-pinene, β-myrcene, β-phellandrene, bornyl acetate, camphene, caryophyllene, caryophyllene oxide, decanal, decanoyl acetaldehyde, 5,4-dioxoaporphines, 2-undecanone, lauraldehyde, limonene, nonanol, n-hexadecanoic acid, oxoaporphines, phytol, α-terpineol, and 4-tridecanone were the main effective components of essential or volatile oils that exhibited a wide range of pharmacological activities [18].

Chou et al. [19] discovered two new compounds in a plu kaow species, houttuynoside A and houttuynamide A. These compounds were found along with 1-decanal, which is responsible for the “fishy” smell of the plant. According to modern studies, decanoyl acetaldehyde is a precursor of methyl n-nonylketone, which may be useful in treating various chronic diseases associated with oxo-inflammation, such as cardiovascular and liver diseases [20,21]. This plant also contains other compounds such as gallic acid, quercitrin, quercetin-3-O-beta-D-galactopyranoside, rutin, resveratrol, chlorogenic acid, which have antioxidant, antidiabetic, anticancer, antihyperlipidemia, and neuroprotective effects [22]. Pharmacological studies have confirmed that plu kaow has a wide range of beneficial effects against allergies, bacterial and viral infections, cancer, chronic sinusitis, fever, neuroinflammatory and immunomodulatory inflammation, rheumatoid arthritis, skin disorders, and immune regulation [17,23]. Additionally, PK might modulate various signaling pathways, gene expression, and cellular metabolism [24]. Wang and Bao [25] demonstrated that houttuynin can significantly alleviate diabetes symptoms by increasing adiponectin levels, which is a crucial factor for insulin sensitivity, and reducing connective tissue growth factors. Meanwhile, Ma et al. [26] found that alkaloids from the aerial part of *H. cordata* exhibit inhibitory activities on protein tyrosine phosphatase 1B (PTP1B), a target for diabetes mellitus treatment, and exhibit hepatoprotective activities. Kumar et al. [27] conducted an in vitro study and found that glucose uptake levels increased compared to insulin. They also hypothesized that this could improve insulin sensitivity. However, previous research has provided some preliminary information about the chemical composition and antidiabetic properties of *H. cordata*, but more detailed structural information about its phytochemical content and the mechanisms underlying these therapeutic activities have not been elucidated. Additionally, the potential of plu kaow ethanolic extract (PK) in treating diabetes and dyslipidemia is not yet fully understood. To address these gaps in knowledge, a study was conducted to explore the untargeted metabolomics and cheminformatics of PK. This study also aimed to investigate its impact on serum glucose levels and various biochemical variables related to carbohydrate, lipid, and protein metabolism. Furthermore, any histological changes in the organs of an animal model were also examined. 

## 2. Materials and Methods

### 2.1. Plant Collection and Preparation of Plu kaow Extract

Fresh aerial plu kaow leaves, stalks, and trunks were collected from an open field at Nam Phong subdistrict, Khon Kaen province, Thailand. Collected plant materials were washed in running water (30–45 min), soaked in tap water (60 min), and finally rinsed with deionized water to remove unwanted residues. Cleaned plant samples were dried at 50 °C for 2–3 days or until constant weight was reached using the oven incubator (BINDER Inc., Bohemia, NY, USA). Oven-dried plant materials were cut into smaller sizes and ground in a blender. To prepare the ethanol extract, the dried finely-ground plant material (20 g) was extracted with 200 mL of ethanol by shaking at 180 rpm, 25 °C for 2 days in the incubator shaker (N-BIOTEK, Gyeonggi-do, Republic of Korea). The clear supernatant was collected by centrifugation at 25 °C, 8000 rpm for 20 min, decanted into a clean round bottom flask, and rotated in a rotary evaporator (ChillSafe, Labogene, Lynge, Denmark) at 45 °C for 1 h. The samples were then freeze-dried at −110 °C (BUCHI, Rotavapor, Denmark) and collected after 48 h. The freeze-dried extract was kept at −80 °C until used.

### 2.2. Chemicals and Reagents

All chemicals and reagents were of analytical grade and purchased from Sigma-Aldrich (St. Louis, MO, USA). The commercial kits for measuring biochemical parameters were also purchased from Sigma-Aldrich and stored at 4 °C.

### 2.3. Total Phenolic Content (TPC)

The total phenolic content of the plu kaow ethanol extract (PK) was determined following Folin and Ciocalteu [28] with minor modifications [29]. In brief, the reaction was assessed in a 96-well plate, where an aliquot part (20 µL) of extract, standard GA, or blank (ethanol) was loaded in each well. Then, the 100 μL of 0.2 M Folin–Ciocalteu reagent and the 80 μL of 7% (*w*/*v*) sodium carbonate were added. The mixtures were incubated at ambient temperature under constant darkness for 30 min. The absorbance value was measured at 760 nm using a microplate reader (Ensight^®^ Multimode Plate Reader, PerkinElmer, Waltham, MA, USA). All detected phenolics were represented as gallic acid equivalents (GAE)/mg dry weight.

### 2.4. Antioxidant Assays

The 2,2-diphenyl-1-picrylhydrazyl (DPPH) and 2,2′-Azinobis-3-ethylbenzotiazoline-6-sulfonic acid (ABTS) assays were used to investigate the radical scavenging activity of PK with some modifications [30]. Gallic acid (Sigma-Aldrich, St. Louis, MO, USA) was used as the reference standard and ethanol was the blank throughout the study.

For the DPPH assay, various concentrations of extracts were mixed with 100 μL of 0.2 mM DPPH reagent and incubated for 30 min under darkness. The reducing power of phytochemicals was evaluated by monitoring the decreased absorbance at 517 nm using the microplate reader (Ensight^®^ Multimode Plate Reader, PerkinElmer, Waltham, MA, USA). The result was expressed as IC_50_ or percentage scavenging of DPPH radical, which could be calculated as follows:

Inhibition ratio (%) = (A_control_ − A_sample_) × 100/A_control_,

where A_control_ = absorbance of the reaction with ethanol and A_sample_ = absorbance of the reaction with extract solution.

For the ABTS assay, the 200 μL of ABTS working solution was mixed well with 10 μL of different concentrations of the PK extract and incubated at room temperature by protecting from the light for 7 min. The decolorization was measured at 734 nm using the microplate reader (Ensight^®^ Multimode Plate Reader, PerkinElmer, Waltham, MA, USA). The obtained result was expressed as IC_50_ and the percentage of radical scavenging activity, which could be calculated according to the formula:

% free radical scavenging = (A_control_ − A_sample_) × 100/A_control_

where A_control_ = absorbance of the reaction with ethanol and A_sample_ = absorbance of the reaction with extract solution.

### 2.5. Detection of Metabolites Using LC-MS/MS

A non-targeted metabolomic (LC-MS/MS) approach was employed to gain more details concerning the phytochemicals in the ethanolic extract. Parameters were based on Wu et al. [17] with appropriate modifications. The analysis was implemented using the ultrahigh-performance liquid chromatograph (UHPLC) (UltiMate 3000 RSLCnano UHPLC System, Thermo Scientific, San Jose, CA, USA) which was equipped with an Acclaim^®^ RSLC120 C18 (100 × 2.1 mm, 2.2 µm 120 Å, Thermo Scientific, San Jose, CA, USA) column. The mobile phases consisted of 0.1% formic acid in water (solvent A) and acetonitrile (solvent B). Elution was performed at 0.4 mL/min using a linear gradient of the solvent B as follows: 2% for 0–1 min, 0–90% for 1–12 min, 90% for 12–14 min, and back to 2% for 4 min, for a total run time of 18 min. The 1 μL of 10 ppm aqueous extract was passed through the column at a temperature of 35 °C. Metabolite identification was confirmed using a mass spectrometer (TripleTOF6600+, AB SCIEX™, Framingham, MA, USA). ESI source conditions were set as follows: the ion source gas 1 50, ion source gas 2 60, curtain gas 30, a temperature of 150 °C, with the ion spray voltage floating of −4500 V. A TOF MS scan range was 100–800 amu., where the product ion scan range was 50–800 Da. The scan accumulation time was 0.2 s, while the parent ion scan accumulation time was 0.25. Mass spectra were generated using a decluttering potential (DP) of −80 V via the collision energy of −40 ± 10 eV. Then, tentative identification of target metabolites was confirmed by comparing their experimental MS/MS spectra with NIST 2017 and Natural Products HR-MS/MS library containing ~13,800 and 1000 substances.

### 2.6. Structural Annotation Using MetFrag Webservice

The raw MS/MS spectra of chosen metabolites from the PK were primarily analyzed using MetFrag software (accessed on 10 April 2024 at https://msbi.ipb-halle.de/MetFrag/) to acquire more details regarding the neutral formula, mass, and possible structure of the query metabolites. This process can be achieved through two steps of data processing: retrieving candidates and performing fragmentation setting and processing. Firstly, a specific formula along with the corresponding *m*/*z* value needs to be provided, where various types of suspected databases (e.g., KEGG, NORMAN, and PubChem) can be chosen to improve the accuracy of annotation. After receiving potential candidates, the experimental MS/MS data was used to match against the in-silico generated spectra of the retrieved candidates. Those achieving an F score closer to or equal to 1.0 should be considered potential candidates for the query subject.

### 2.7. Metabolite Annotation Using Sirius (v. 5.6.)

The same raw mass data set of PK’s metabolites was imported into the software. The MS2 level was set to a collision-induced dissociation (CID) energy of (30–50) eV, with the precursor ion (e.g., *m*/*z* 447.1004) and adduct type specified. During this step, the expected formula (e.g., C_21_H_20_O_11_) was sometimes used to enhance the specificity of the substance annotation. Upon clicking ‘compute’, Sirius, CSI:FingerID, and CANOPUS were chosen to comprehend more details of natural products. The inclusion of biological databases such as KEGG, NORMAN, Plantcyc, and Natural Products helps improve the accuracy of metabolite annotation.

### 2.8. In Silico Analysis

The seven target proteins chosen to explain plausible mechanisms underlying the in vivo antidiabetic activity of the PK were based on Van et al. [31], with some modifications. These proteins included alpha-amylase (PDB: 1SOE), alpha-glucosidase (PDB: 1UOK), the sulfonylurea receptor (SUR, PDB: 2E5Z), glycogen phosphorylase (GP, PDB: 1NOI), glucagon-like peptide−1 (GLP1, PDB: 3IOL), insulin-like growth factor 1 receptor kinase (IGF1R, PDB: 1K3A), and peroxisome proliferator-activated receptor gamma (PPAR-γ, PDB: 5YCP). CB-Dock2 (accessed on 10 April 2024 at https://cadd.labshare.cn/cb-dock2/php/blinddock.php#job_list_load) is used to generate complex structures of α-glucosidase/acarbose, SUR/luteolin, GLP-1/metformin, GP/NTZ, IGF1R/glibenclamide, and PPAR-γ/rosiglitazone as templates for analysis with GOLD 5.2.2 (Genetic Optimization of Ligand Docking). After self-docking was performed, all seven receptor-ligand complex structures exhibited RMSD values less than 2.0 Å, indicating their suitability for docking purposes. “The docking parameters, such as binding sites and scoring functions (e.g., CHEMPLP, GoldScore, ChemScore, and ASP), were optimized depending on the specific types of target proteins. The fitness score, as determined by the GOLD software, is defined as the negative sum of the component energies, in which a larger (less negative) fitness score indicates a better binding energy.

### 2.9. Hemolytic Activity of Plu kaow Ethanolic Extracts

The hemolytic activity of the extracts was assessed as described by Alinezhad et al. [32]. Erythrocytes from blood were separated by centrifugation and washed with phosphate buffer (pH 7.4). Erythrocytes were then diluted with phosphate-buffered saline to give 4% suspension. Then, 0.5 mL of different concentrations of each extract was added to 2 mL of erythrocyte suspension and the volume was made up to 5 mL with saline buffer. The mixture was incubated for 5 min at room temperature and then 0.5 mL of hydrogen peroxide solution in saline buffer was added to induce the oxidative degradation of the membrane lipids. The concentration of hydrogen peroxide in the reaction mixture was adjusted to bring about 90% hemolysis of blood cells after 240 min. After incubation, the reaction mixture was centrifuged at 250× *g* for 10 min and the extent of hemolysis was determined by measuring the absorbance at 540 nm corresponding to hemoglobin liberation.

### 2.10. Animals

Healthy Long Evans male rats (160–210 g) were acquired from the Animal House, Faculty of Biological Sciences, Islamic University, Bangladesh. Experimental animals were familiarized for a week prior to starting the experimentation, and normal chow was given. A standard environmental condition (24 ± 2 °C, 45 ± 5% humidity, and a 12-h light/dark cycle) was maintained with free access to drinking water.

### 2.11. Diet for the Animals

A standard laboratory diet (20% rice polish, 21% wheat bran, 30% wheat flour, 10% protein source/fishmeal, 10% oilseed cake, 5% molasses, 2% soybean oil, 0.5% vitamin, and 1.5% common salt) proposed by Rahman et al. [33] was given daily to all groups of rats.

### 2.12. Experimental Design

To evaluate the effect of plu kaow ethanolic extract (PK) on hyperglycemia and dyslipidemia in normal and STZ-induced diabetic rats, the animals were divided into five groups (*n* = 8), as follows:
Group 1:Untreated healthy control rats (HC)Group 2:Untreated diabetic control rats (DC)Group 3:Healthy rats treated with PK ethanolic extract (500 mg/kg/day in 1 mL water) (HPK)Group 4:Diabetic rats treated with PK ethanolic extract (500 mg/kg/day in 1 mL water) (DPK)Group 5:Diabetic rats treated with glibenclamide (5 mg/kg/day in 1 mL water) (DD)

Ethanolic extract of plu kaow (500 mg/kg/rat/day) was dissolved in 1 mL distilled water as suspension and orally fed daily to the experimental rats (groups 3 and 4) through a feeding syringe in addition to the regular diet. The untreated healthy and diabetic rats (groups 1 and 2) were administered drinking water daily, and group 5 was given glibenclamide as a reference drug (5 mg/kg/day). The investigation of this study was recorded regularly and continued for 28 consecutive days.

### 2.13. Induction of Diabetes in Rats

**Figure 1:**
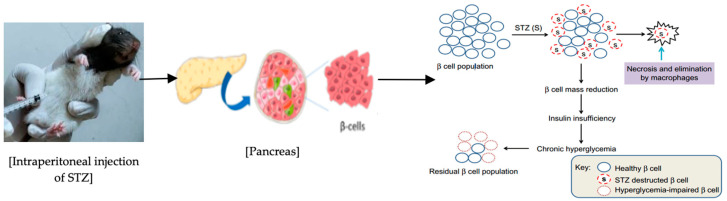
Mechanism of pancreatic beta-cells damage with intraperitoneal injection of streptozotocin [36].

Diabetes was induced in overnight fasted Long Evans rats by injecting (intraperitoneally) a single dose of freshly prepared streptozotocin (STZ) (Sigma, St. Louis, MO, USA) at a dose of 65 mg/kg body weight (b.w) dissolved in 0.1 M cold sodium citrate buffer with pH 4.5 [34]. The non-diabetic control rats (groups 1 and 3) also received an injection of the citrate buffer. After 3 days of STZ administration, diabetes was induced by destroying the beta cells (as shown in Figure 1). Tail blood samples of the overnight fasted rats were collected to measure blood glucose levels. Diabetic rats with fasting blood glucose (FBG) levels higher than 250 mg/dL (>13.8 mmol/L) were used for the experiments [35]. Both diabetic and non-diabetic rats were kept separately in metabolic cages.

### 2.14. Collection of Blood and Determination of Biochemical Parameters

Blood samples were collected from the tail vein of the animals after 12 h of fasting on days 0, 7, 14, 21, and 28. Glucose levels were determined using glucose oxidase/peroxidase reactive strips and a glucometer (Accu Check Advantage^®^, Roche Diagnostics, Mannheim, Germany). At the end of the treatment, the animals were fasted overnight, and blood samples were drawn from their retro-orbital plexus. The serum was immediately isolated from the blood samples by centrifugation at 3000× *g* rpm for 10 min and analyzed for various biochemical parameters. The serum samples were stored at −80 °C in a freezer until they were analyzed. Biochemical parameters such as serum cholesterol, triglycerides, HDL-cholesterol, LDL-cholesterol, VLDL-cholesterol, serum glutamic oxaloacetic transaminase (SGOT), serum glutamic pyruvic transaminase (SGPT), alkaline phosphatase (ALP), serum creatinine, and cardiovascular enzymes (CK and CKMB) were measured using standard methods [37] and diagnostic kits with an auto biochemistry analyzer (BIOELAB AS-280, Dhaka, Bangladesh). 

### 2.15. Histological Investigation

A 10% neutral buffered formalin solution was used to fix the pancreas, liver, kidneys, spleen, and heart from the experimental animals after sacrifice and dissection. These fixed organs were transferred to the Pathology department, Doctors Lab, and Hospital (Pvt.) Ltd., Kushtia, Bangladesh for further processing, sectioning, and staining. All tissues were sliced at a thickness of 5 mm and embedded in paraffin. A modified aldehyde fuchsin method was used to stain pancreatic sections, whereas hematoxylin and eosin (H&E) were used to stain other organ sections [38]. Photomicrographs of various organs were captured using a confocal microscope (Model: Ti2-E Nikon, Tokyo, Japan).

### 2.16. Statistical Analysis

The data were analyzed using SPSS version 24 (SPSS/IBM, Chicago, IL, USA). The results were expressed as the mean ± standard deviation (SD). One-way analysis of variance (ANOVA) was used, and paired or unpaired *t*-test was performed for multiple comparisons between groups. A value of *p* < 0.05 was considered statistically significant, while *p* < 0.001 was considered highly significant.

### 2.17. Ethical Issues

This study was conducted in accordance with standard ethical guidelines of the Institutional Animal Ethical Committee at the Faculty of Biological Science, Islamic University, Bangladesh. The Institutional Review Board of the Islamic University approved this study (Reference no.: AWEEC/FBS/IU/2023/02).

## 3. Results

### 3.1. Effect of Plu kaow on Hyperglycemia of Diabetic and Normal Rats

**Figure 2:**
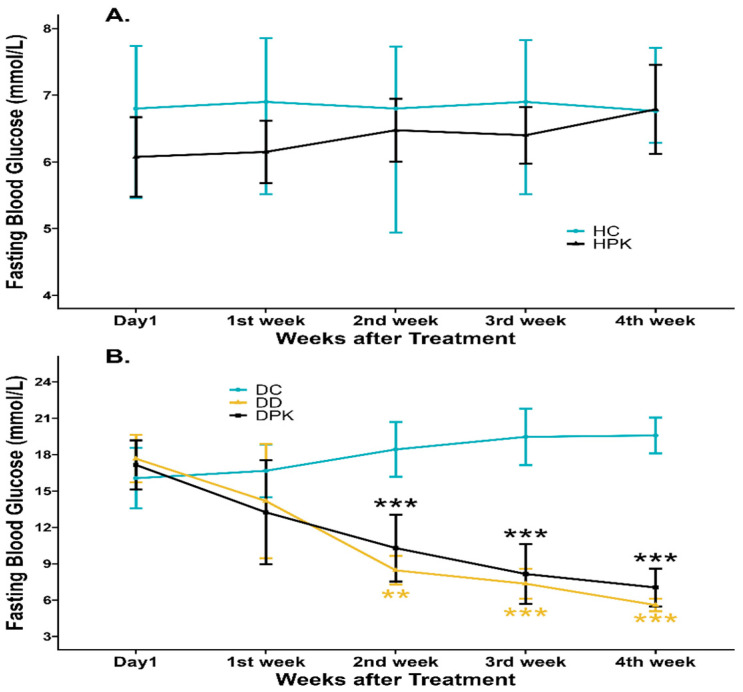
Fasting blood glucose levels of different intervention groups (*n* = 8) at each time point ((**A**). Intervention in healthy groups, (**B**). Intervention in diabetes groups). Here, HC: healthy control; HPK: healthy rats treated with plu kaow; DC: diabetic control; DD: diabetic rats treated with glibenclamide; and DPK: diabetic rats treated with plu kaow [Results presented as mean with 95% CI, Output from repeated measure of ANOVA and *p* value indicates significant difference of blood glucose level at each time points when compared with Day 1. **: *p* < 0.01, ***: *p* < 0.001.

The data depicted in Figure 2 indicates that blood glucose levels were significantly higher (*p* < 0.05) in diabetic control rats (Group 2) compared to the other groups. Following the administration of PK at a dose of 500 mg/kg/day for 28 days, diabetic rats (Group 4) showed a significant (*p* < 0.001) reduction in glucose levels, approaching normal levels as compared to untreated diabetic rats (Group 2). These findings are consistent with the results reported by Kumar et al. [39], who administered the extract at doses of 200 and 400 mg/kg daily for 21 days. However, when healthy non-diabetic rats (Group 3) received the same treatment, the serum glucose concentration did not significantly differ compared to untreated healthy rats (Group 1), as illustrated in Figure 2.

### 3.2. Evaluation of the Effect of Plu kaow Ethanolic Extract on Dyslipidemia

Table 1 shows the biochemical parameters, such as total cholesterol (TC), triglycerides (TG), low-density lipoprotein (LDL), very low-density lipoprotein (VLDL), and high-density lipoprotein (HDL) levels. In the STZ-induced diabetic rats, the levels of TC, TG, LDL, and VLDL were significantly raised (*p* < 0.001) and HDL decreased compared to all other groups. However, treatment of diabetic rats with PK (Group 4) reduced (*p* < 0.001) these elevated levels of TC, TG, LDL, and VLDL-cholesterol to values comparable to those of healthy rats (Group 1) and positive control group (Group 5). Moreover, there was a significant elevation (*p* < 0.001) in HDL-cholesterol observed in diabetic rats treated with PK, as seen in Table 1. In contrast, there were no significant changes in the levels of lipid profile in normal treated rats (Group 3).

The ratio of total cholesterol (TC) to high-density lipoprotein (HDL) and low-density lipoprotein cholesterol (LDL) to HDL is a simple and useful index to determine the risk of ischemic heart disease. A higher ratio indicates a higher risk of heart disease. This ratio is a relevant cumulative marker of metabolic abnormalities, which are typically found in individuals with high triglycerides and low HDL cholesterol levels. This condition is often caused by abdominal obesity and insulin resistance and is associated with an increased concentration of small, dense LDL particles. A significantly (*p* < 0.001) reduced ratio of TC/HDL and LDL/HDL in this study suggests that PK can lower the risk of ischemic heart disease (Table 1).

### 3.3. Effect of Plu kaow Ethanolic Extract on Hepatic, Cardiac, and Renal Function Markers Activity

Table 2 shows the changes in the activity of three enzymes (SGPT, SGOT, and ALP) in the serum of rats after being treated with PK for 28 days. The study found that untreated diabetic rats had significantly higher levels of these enzymes compared to other groups (*p* < 0.05). However, treatment with PK reduced these high enzyme levels to values that were similar to those in healthy control rats (Group 1).

**Figure 3:**
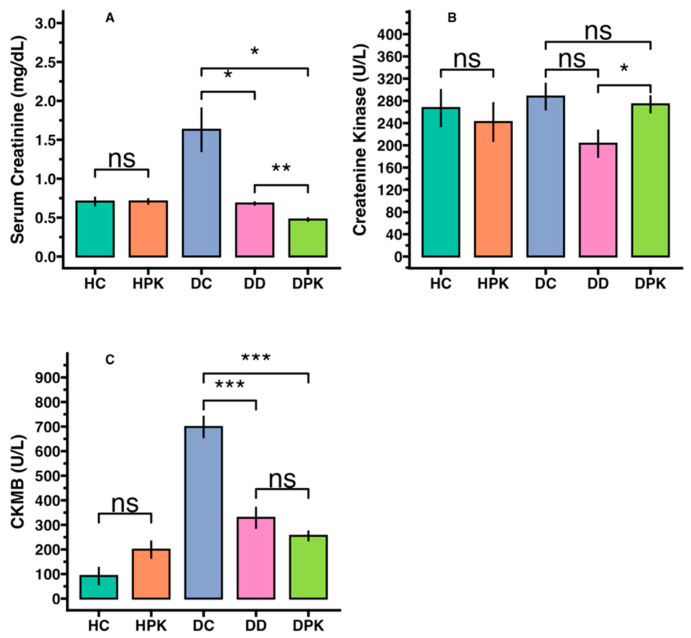
Evaluation of the effect of plu kaow ethanolic extract on cardiovascular, and renal function markers. Values are expressed as mean ± SD, (*n* = 8). HC (Healthy control); HPK (Healthy treated group); DC (Diabetic control); DPK (Diabetic treated group); DD (Diabetic drug group). Here, (**A**) indicates renal marker; (**B**,**C**) indicate cardiovascular markers; * *p* < 0.05, ** *p* < 0.01, *** *p* < 0.001, ns = non-significant.

Figure 3 illustrates the outcomes of cardiac and renal function markers across all experimental groups. In diabetic rats, the activities of cardiac function markers (CK and CKMB) and renal function markers (serum creatinine) significantly increased (*p* < 0.001). However, in the diabetic-treated group (Group 4), the raised enzymatic activities returned to normal levels. A similar effect was observed in the glibenclamide-treated rats (Group 5). Conversely, there were no significant changes in the levels of cardiac and renal function markers in the normal-treated rats (Group 3).

### 3.4. Effect of Plu kaow Extract on Body Weights and Food Consumption

**Figure 4:**
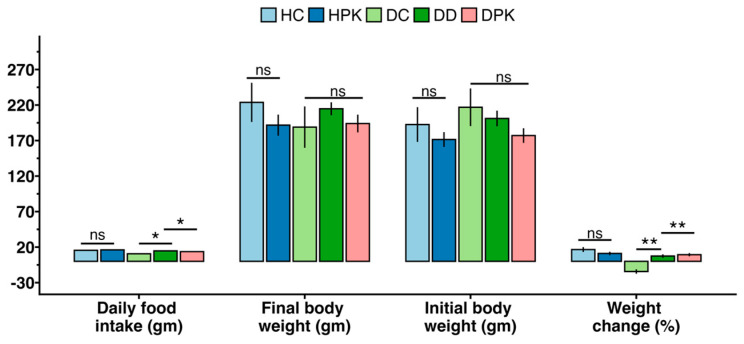
Body weight and food intake in both healthy and diabetic groups. Values are expressed as mean ± SD, (*n* = 8). HC (Healthy control); HPK (Healthy treated group); DC (Diabetic control); DPK (Diabetic treated group); DD (Diabetic drug group); * *p* < 0.05, ** *p* < 0.01 and ns = non-significant.

**Figure 5:**
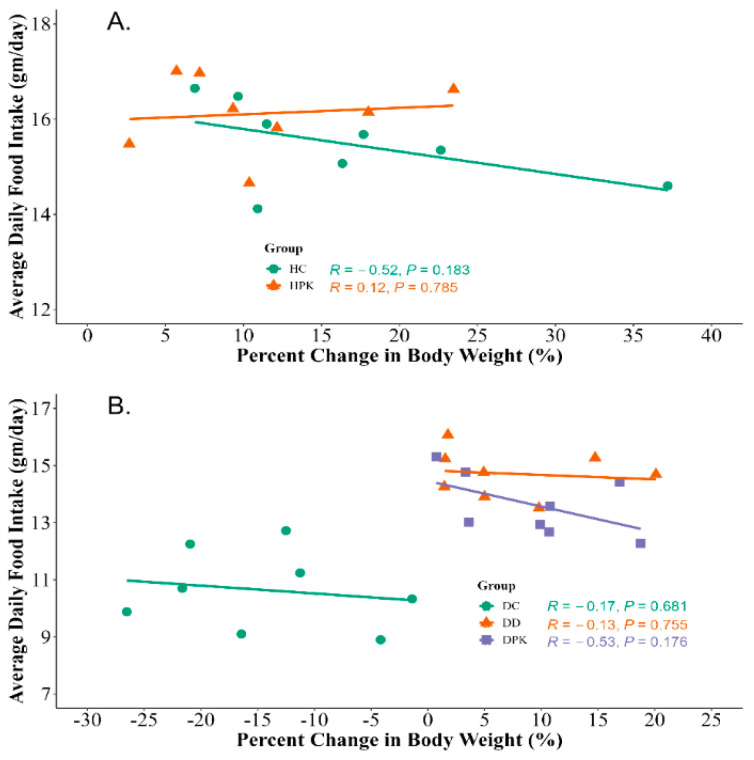
Correlation between average daily food intake and percent change in body weight. Subfigures label (**A**) indicates intervention in healthy group and label (**B**) indicates intervention in diabetic group; HC (Healthy control); HPK (Healthy treated group); DC (Diabetic control); DPK (Diabetic treated group); DD (Diabetic drug group).

During the study, it was found that the body weight of diabetic rats decreased significantly (*p* < 0.001) when compared to the healthy control group (Group 1). However, the rats treated with 500 mg/kg/day of PK and 5 mg/kg/day of glibenclamide showed a restoration of their final body weight to the baseline level, similar to that of the control group. However, no significant changes were found in the case of non-diabetic treated rats (Group 3). The same changes were also noticed in food consumption among the groups as shown in Figure 4. The correlation between the average daily food intake and the percent change in body weight was also presented in Figure 5.

### 3.5. Effect of PK Extract on Organs Relative Weight of Liver, Kidney, Heart, Spleen, and Pancreas

**Figure 6:**
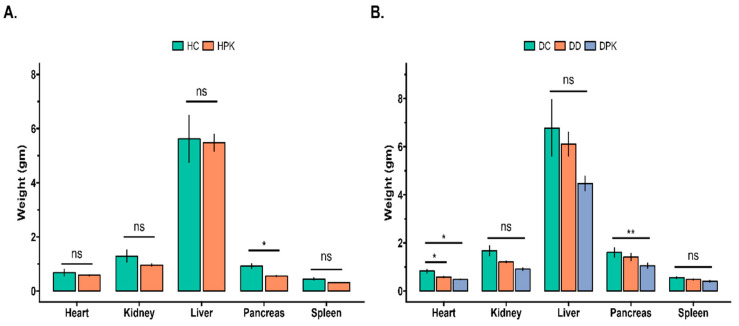

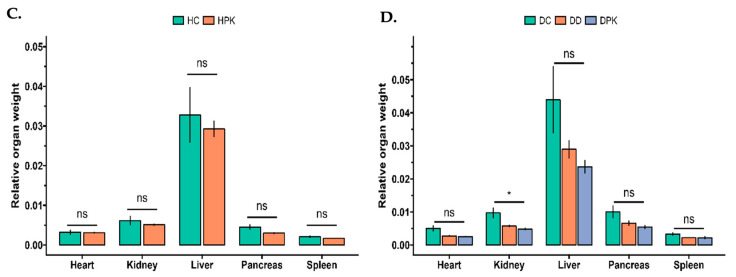
Organ weight and relative organ weight (organ/body weight) in both healthy and diabetic groups. Subfigures label (**A**,**C**) indicates intervention in healthy groups and Subfigures label (**B**,**D**) indicates intervention in diabetic groups. Here, HC (Healthy control); HPK (Healthy treated group); DC (Diabetic control); DPK (Diabetic treated group); DD (Diabetic drug group). Output from repeated measure of ANOVA and *p* value indicates significant difference between two groups at each time point. * *p* < 0.05, ** *p* < 0.01 and ns = non-significant.

There were no significant differences in the weight of the various organs among all the groups, except the pancreas (as shown in Figure 6). The rats treated with PK (Groups 3 and 4) had a significantly reduced weight of the pancreas (*p* < 0.01) compared to the corresponding control groups. It is not clear why pancreas weight is reduced in treated groups, so it should be explored in further research. Both the positive control group (Group 5) and the diabetic-treated group (Group 4) had a reduced heart weight of 29.92% and 42.11%, respectively, compared to the diabetic control rats (Group 2). In groups 2 and 4, the mean value of liver weight was 6.8 ± 1.2 and 4.5 ± 0.3 g, while kidney weight was 1.7 ± 0.2 and 1.2 ± 0.1 g, respectively.

This study also investigated the relative organ weights (organ/body weight) of the rats (as shown in Figure 6). The administration of PK or glibenclamide did not significantly (*p* < 0.05) affect the organ weights of the rats compared to the corresponding control rats.

### 3.6. Histopathological Examination of Various Organs of Experimental Rats

**Figure 7:**
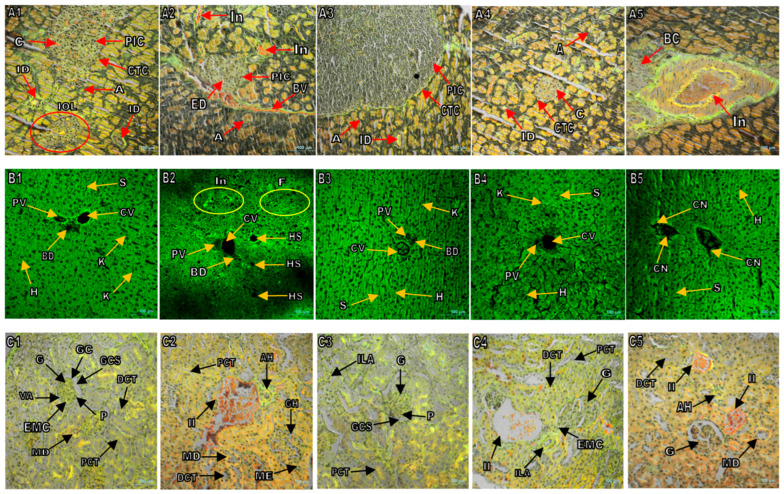

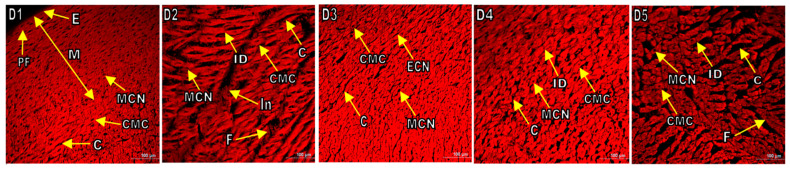
(**A1**–**A5**) Histopathological changes in pancreatic cells treated with ethanolic extract of plu kaow. The photomicrograph was captured by a Confocal Microscope with an object size of x40 and a 100μm scale bar (Mod: Ti2-E Nikon). The section of the pancreas of the following groups was compared: (**A1**) normoglycemic control, (**A2**) diabetic control showing extensive damage to the islets and reduced dimensions of islets, (**A3**) healthy rat treated with plu kaow, (**A4**) diabetic rat treated with plu kaow (500 mg/kg b.w./day), and (**A5**) diabetic rat treated with glibenclamide. The components identified in the photomicrograph are C (Capillaries), PIC (Pancreatic Islet Cell), CTC (Connective Tissue Capsule), A (Acini), ID (Interlobular Duct), IOL (Islet of Langerhans), In (Inflammation), ED (Extensive Damage), BV (Blood Vessel), BC (Beta Cell). It was observed that the diabetic rats treated with plu kaow extract showed significant restoration of the size of islets and beta-cells, and regenerated Islet of Langerhans. (**B1**–**B5**) Histopathological changes in hepatic cells treated with ethanolic extract of plu kaow. The photomicrograph was captured by a Confocal Microscope with an object size of x40 and a 100μm scale bar (Mod: Ti2-E Nikon). Here, (**B1**) normoglycemic control, (**B2**) diabetic control showing extensive damage to the liver cells, (**B3**) healthy rat treated with plu kaow, (**B4**) diabetic rat treated with plu kaow (500 mg/kg b.w./day), and (**B5**) diabetic rat treated with glibenclamide. The components identified in the photomicrograph are CV (Central Vain), PV (Portal Vain), Bd (Bile Duct), K (Kuffer Cell), In (Inflammation), S (Sinusoid), H (Hepatosite), HS (Hepatic Steatosis), F (Fibrosis), CN (Centrilobular Necrosis). Diabetic rats treated with plu kaow extract (500 mg/kg/day) showed marked improvement of central vein (CV) and sinusoid (S). (**C1**–**C5**) Histopathological changes in kidney cells treated with ethanolic extract of plu kaow. The photomicrograph was captured by a Confocal Microscope with an object size of x40 and a 100μm scale bar (Mod: Ti2-E Nikon). Here, (**C1**) normoglycemic control, (**C2**) diabetic control (**C3**) healthy rat treated with plu kaow, (**C4**) diabetic rat treated with plu kaow (500 mg/kg b.w./day), and (**C5**) diabetic rat treated with glibenclamide. G (Glomerulus), GC (Glomerulus Capsule), GCS (Glomerulus Capsular Space), DCT (Distal Convoluted Tubule), PCT (Proximal Convoluted Tubule), EMC (Extraglomerular Mesangial Cell), ILP (Interlobar Arteries), MD (Macula Densa), VA (Vascular Pole), *p* (Podocyte), AH (Arteriolar Hyalinosis), II (Interstitial Inflammation), GH (Glomerular Hypertrophy), ME (Mesangial Expansion). Diabetic rats treated with plu kaow extract showed marked improvement in Interstitial Inflammation (II), Glomerular Hypertrophy (GH), and Arteriolar Hyalinosis (AH). (**D1**–**D5**) Histopathological changes in heart cells treated with ethanolic extract of plu kaow. The photomicrograph was captured by a Confocal Microscope with an object size of x40 and a 100μm scale bar (Mod: Ti2-E Nikon). Here, (**D1**) normoglycemic control, (**D2**) diabetic control (**D3**) healthy rat treated with plu kaow, (**D4**) diabetic rat treated with plu kaow (500 mg/kg b.w./day), and (**D5**) diabetic rat treated with glibenclamide. Here, E (Endocardium), M (myocardium), PF (Purkinje Fiber), MCN (Muscle Cell Nucleus), CMC (Cardiac Muscle Fiber), C (Capillary), F (Myocardial Fibrosis), In (Myocardial Inflammation), ENC (Endothelial Cell Nucleus), ID (Intercalated Discs). Diabetic rats treated with plu kaow (500 mg/kg body weight/day) showed marked improvement in Myocardial Inflammation (In), Myocardial Fibrosis (F).

Histopathological analysis of pancreatic tissues from normal, PK-treated, and drug-treated rats are presented in Figure 7A1–A5. The confocal micrographs of the pancreatic islet area of healthy control (HC) rats revealed compact acinar cells with ample granular eosinophilic cytoplasm and normal inter-lobular connective tissues with intact islets of Langerhans (Figure 7A1). In contrast, STZ-induced diabetic rats showed degeneration and necrotic changes in the pancreatic islets’ cells with shrunken cells (Figure 7A2). Pancreatic sections from the PK-treated groups (500 mg/kg/day) showed a partial regeneration of the pancreatic islets, as seen in Figure 7A4. The pancreas of the glibenclamide-treated group appeared almost identical to the diabetic control group (Figure 7A5).

Microscopic images of hepatic tissues were also examined and compared among different groups of rats. The first group consisted of normal rats, while the second group had diabetic rats. The images showed that normal rats had well-arranged hepatic cells radiating from a normal hepatic central vein (Figure 7B1). However, diabetic rats had severe hydropic degeneration with cloudy and fatty cells, along with inflammatory cell infiltration (Figure 7B2). On the other hand, the third group of rats, which were non-diabetic and treated with PK, showed no significant pathological changes in the hepatic tissue (Figure 7B3). Finally, diabetic rats treated with PK and glibenclamide showed a significant improvement in the precipitated lesions and had no inflammatory infiltration (Figure 7B4,B5).

The following observations were also made in the study: kidney tissues of the healthy control rats (Group 1) appeared normal with healthy glomeruli and tubules (as shown in Figure 7C1). In contrast, the diabetic control rats (Group 2) showed shrunken, fragmented, and some lysed glomeruli, thickened tubules, and tubular necrosis (Figure 7C2). The healthy treated group (Group 3) showed no changes in kidney tissues (Figure 7C3). The diabetic group treated with PK showed mostly normal kidney tissues with healthy glomeruli and tubules, and no inflammatory cells were detected in the image of kidney tissue (Figure 7C4). On the other hand, the glibenclamide-treated group showed a moderate degree of tissue damage with less tubular necrosis and fewer fragmented/shrunken glomeruli. No inflammatory cell infiltration was detected (as shown in Figure 7C5).

The healthy heart tissues in rats showed normal myocardium with regular myofibrils and muscle bundles, as seen in Figure 7D1. However, in diabetic rats, the structure of myofibrils was disorganized, and muscle bundles were detached due to necrosis (Figure 7D2). In the case of healthy treated rats, a slight inflammatory infiltration was detected (Figure 7D3). On the other hand, diabetic rats treated with PK extract showed mostly normal myocardial muscle bundles and myofibrils, as compared to the pathological improvement of lesions on the heart tissue of drug-treated rats (Group 5), as shown in Figure 7D4,D5.

### 3.7. Hemolytic Activity of Plu kaow Ethanolic Extract

**Figure 8:**
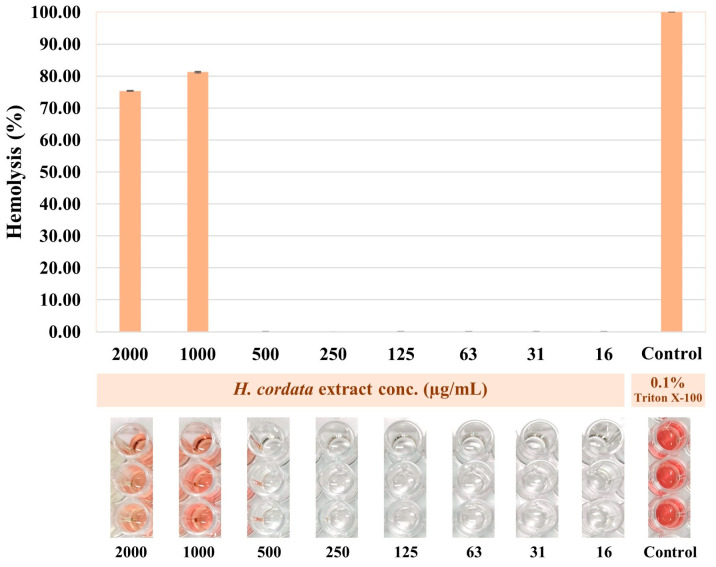
Hemolytic activity of plu kaow ethanolic extract.

The hemolytic activity of PK was tested on normal human erythrocytes, and the extracts did not exhibit hemolytic activity at concentrations ranging from 16–500 µg/mL, but it showed a clearly distinctive effect at concentrations between 1000–2000 µg/mL, without a dose-dependent pattern, as seen in Figure 8. This study is the first to report on the hemolytic activity of plu kaow (*H. cordata*) ethanolic extract.

### 3.8. Antioxidant Activity of Plu kaow Ethanolic Extract

The antioxidant activity of the PK was determined using the DPPH assay, ABTS, and total phenolic content. The results showed that PK had better scavenging activity, with IC_50_ values of ABTS (0.44 mg/mL) and DPPH (0.16 mg/mL), compared to gallic acid and quercetin (Table 3). The IC_50_ values for gallic acid and quercetin were 0.03 and 0.04 mg/mL for ABTS and 0.003 and 0.018 mg/mL for DPPH, respectively, while the total phenolic content of PK was estimated to be 84.87mg (GAE)/g.

### 3.9. Molecular Docking

The possible mechanism behind the reversal of hyperglycemia is quite intricate, involving various pathways and target proteins. These include α-amylase, α-glucosidase, sulfonylurea receptor (SUR), glycogen phosphorylase (GP), glucagon-like peptide-1 (GLP-1), insulin-like growth factor 1 (IGF1R) kinase, and peroxisome proliferator-activated receptor-gamma (PPAR-γ) [40].

In the intestines, inhibiting the carbohydrate-degrading enzymes α-amylase and α-glucosidase reduces glucose absorption. It delays its release into the bloodstream, thus improving conditions such as type-2 diabetes mellitus (T2DM) [41]. Alternatively, increasing cellular calcium concentrations by inhibiting SUR also enhances the incorporation of insulin granules into membrane regions, subsequently improving glucose uptake due to enhanced insulin secretion into the plasma [42]. In addition to enhancing glucose uptake/utilization and controlling lipid metabolism and protein synthesis, IGF1R also contributes to hormonal balance by regulating various hormones in the body (e.g., growth hormone (GH) and insulin), thereby alleviating diabetic conditions. Activation of this protein can improve insulin sensitivity, reduce insulin requirement, and enhance glycemic control in severe cases, such as insulin-resistant syndromes [43]. Furthermore, inhibiting glycogen phosphorylase (GP) is a promising approach for treating Type 2 Diabetes Mellitus (T2DM), as it impedes glycogenesis and reduces hepatic glucose production, resulting in decreased blood glucose levels [44]. GLP-1 agonists such as exenatide, liraglutide, lixisenatide, and taspoglutide have emerged as crucial treatments for non-insulin-dependent diabetes mellitus due to their positive effects like sulfonylureas (SUR) [45]. Additionally, the activation of peroxisome proliferator-activated receptor-gamma (PPAR-γ) by certain drugs (e.g., thiazolidinediones) not only enhances the uptake of fatty acids from the bloodstream into adipose tissues but also improves insulin sensitivity through cellular responses [46].

Based on a metabolomics study in conjunction with computational-based analyses, the plu kaow ethanolic extract (PK) has been fully analyzed, and structurally annotated to contain a substantial number of flavonoids and other glycosylated derivatives. The plausible molecular mechanisms underlying the antidiabetic activity of certain simple phenolics, flavonoids, and their glycosylated analogs (as shown in Table 4) in rat models were investigated to determine if they are presumed to be highly relevant to the observed in-silico pharmacology of the plant extract. To accomplish this, several antidiabetic receptors, including α-amylase, α-glucosidase, SUR, GP, GLP-1, IGF1R, and PPAR-γ, were examined in molecular docking studies. The in-silico analysis revealed that the query ligands were predicted to exhibit antidiabetic activity by targeting multiple receptors, characterized by binding energies higher than those of conventional antidiabetic drugs (e.g., rosiglitazon, acarbose, and glicazide) (Table 4). For instance, rutin demonstrated strong interactions with all tested receptors, illustrating the polypharmacological effects of this phenolic compound. Furthermore, many natural products displayed high binding energies, with slight differences from those observed in standard antidiabetic drugs, suggesting their potential efficacy as active agents. However, to be considered active agents, these compounds must specifically interact with each receptor’s active site and drug recognition region by forming hydrogen and electrostatic bonds, maintaining distances within 3.00 Å and 5.00 Å, respectively [47]. The flavonoids detected in the PK are expected to regulate hyperglycemic conditions through specific and simultaneous interactions with the selected targets. Detailed characteristics of these interactions are provided below:

#### 3.9.1. Alpha-Amylase

**Figure 9:**
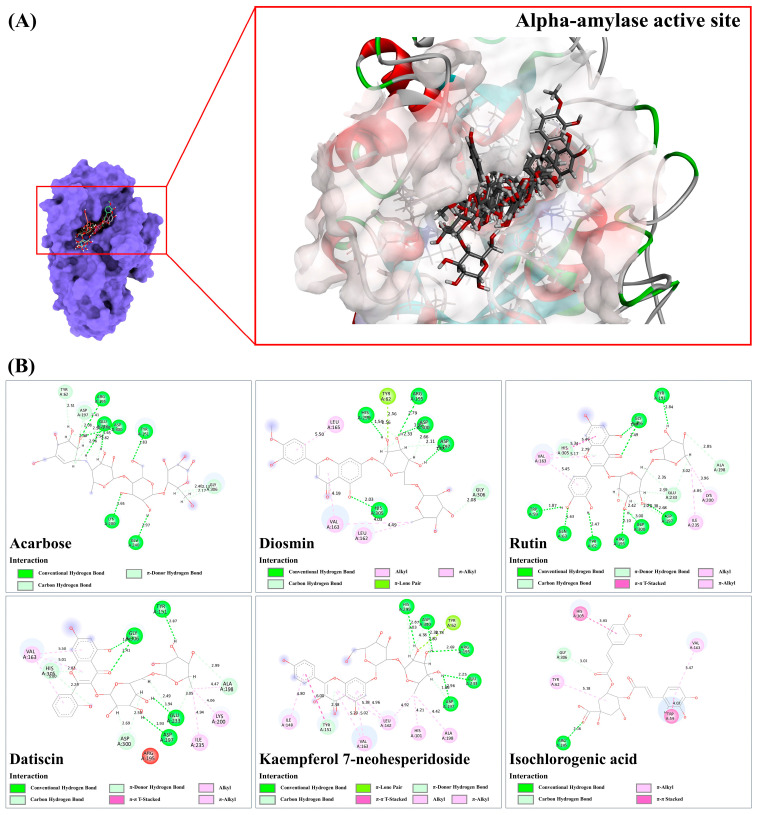
(**A**,**B**) showed 3D and 2D representations of the interaction of acarbose, diosmin, rutin, datiscin, kaempferol 7-neohesperidoside, and isochlorogenic acid with α-amylase.

In accordance with the criteria, five of the 21 docked metabolites, specifically kaempferol 7-neohesperidoside, isochlorogenic acid, rutin, datiscin, and diosmin, were deemed active due to their stronger binding energy compared to the reference drug acarbose, which had a fitness score of 66.44. This glycemic control agent formed nine hydrogen bond interactions with various active residues, including Tyr151, Arg195, Lys200, Glu240, and Asp300, with bond lengths ranging between 1.65 and 2.97 Å in the α-amylase structure. Additionally, it weakly formed carbon-hydrogen and π-hydrogen bond interactions with the same and adjacent amino acids, including Tyr62, Asp197, Glu233, Asp300, and Gly306 (2.08–2.96 Å). Among these, diosmin had the highest fitness score (75.28), revealing five conventional hydrogen bonds (1.54–2.79 Å) with polar amino acids, including Arg195, Asp197, His299, Asp300, and His305, located in the α-amylase target. Its methoxy group (-OCH_3_) attached to the sugar moiety and aromatic system (rings B and C) was further stabilized by forming alkyl-alkyl and π-alkyl interactions with Leu162, Val163, and Leu165 (4.03–5.50 Å). Additionally, five C-H interactions (2.08–2.66 Å) with Asp197, His299, Asp300, and Gly306, along with one π-lone pair interaction with Tyr62 (2.56 Å), were also established via its sugar moiety. Rutin and datiscin also exhibited binding capacities like those observed with the reference drug, with binding scores of 70.95 and 71.30, respectively. In the active site of α-amylase, nine hydrogen bonds were established with Trp55, Tyr62, Gln63, Tyr151, Arg195, Asp197, Asp300, and Gly306, with bond lengths ranging between 1.50 and 3.00 Å, thus fulfilling the established criteria. Rutin, like acarbose, engaged in carbon-hydrogen and π-hydrogen bonding interactions with both identical and adjacent amino acids, such as His305 and Ala198, with bond distances spanning from 1.96 to 3.02 Å. It also formed six robust hydrophobic interactions, including alkyl-alkyl, π-alkyl, and π-π T-shaped interactions with Val163, Lys200, and Ile235, ranging from 3.96 to 5.46 Å, which were remarkably absent in the reference α-amylase antagonist. Similarly, datiscin well established five hydrogen bond interactions with Tyr151, Asp197, Glu233, and Gly306, while C-H and π-hydrogen bond interactions were also observed in the target site, with bond lengths ranging between 2.29 and 2.99 Å. Moreover, it reveals hydrophobic interactions, such as alkyl, π-alkyl, and π-π T-shaped, with Val163, Ala198, Lys200, and Ile235, within the same region, ranging from 4.06 to 5.50 Å. Kaempferol 7-neohesperidoside, which is predicted to localize in the druggable target, forms five hydrogen bond interactions with five polar active residues, including Arg195 (2.69 Å), Asp197 (2.96 Å), Glu233 (2.25 Å), His299 (2.87 Å), and Asp300 (2.38 Å). It primarily utilizes hydroxyl groups, belonging to the glycoside moiety, to simultaneously establish C-H, π-donor H, and π-lone pair interactions with the aforementioned amino acid residues (ranging from 1.86 to 3.03 Å). Furthermore, its kaempferol core structure (comprising benzene rings A and B, and heterocyclic ring C) and the methyl group (-CH_3_) gently form numerous hydrophobic interactions with the active residues, including His101, Ile148, Tyr151, Leu162, Val163, and Ala198 (4.21 to 6.0 Å). Isochlorogenic acid C, with a binding score of 69.77, forms five hydrogen bond interactions with His101 (2.74 Å), Arg195 (6.37 and 1.53 Å), Asp197 (2.00 Å), and Glu233 (1.99 Å). Additionally, its caffeic acid-derived moiety establishes a π-π stacked interaction with Trp59 (3.48 Å), resulting in a strong interaction with the amylase active site (Figure 9).

#### 3.9.2. Alpha-Glucosidase

**Figure 10:**
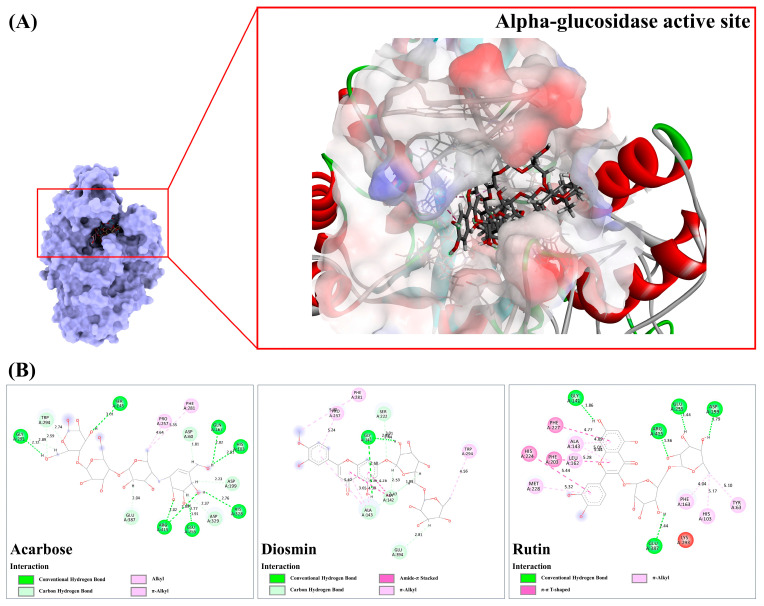
(**A**,**B**) showed 3D and 2D representations of the interaction of acarbose, diosmin, and rutin with α-glucosidase.

Upon docking against the alpha-glucosidase structure, it was observed that only two metabolites, diosmin, and rutin, displayed higher binding energies compared to the reference drug, acarbose (49.78). In our investigation, the anti-diabetic drug demonstrated affinity by establishing eight hydrogen bond interactions with seven active residues, including His103, Ser145, Gln167, Glu255, Gly291, His328, and Arg415, with bond lengths ranging from 1.91 to 3.01 Å. It also participated in multiple C-H bond interactions with adjacent amino acids (Asp60, Asp199, Gly291, Trp294, and Asp392), demonstrating bond lengths ranging from 1.81 to 2.89 Å. Additionally, only two hydrophobic interactions (4.64–5.35 Å) were deduced (Figure 10). In contrast, diosmin exhibited two robust hydrogen bonds with Gly141 (2.44–2.58 Å), along with the detection of five C-H bond interactions in the target region (1.99–3.01 Å). By predominantly utilizing the basic flavone skeleton, it demonstrated the capability to form a significant number of alkyl-alkyl and amide-π stacked interactions with five amino acid residues, namely Ala142, Ala143, Pro257, Phe281, and Trp294 (3.65 and 5.30 Å). As compared with acarbose, rutin formed only four hydrogen bond interactions with Gly141, Asp199, Glu387, and Arg415 (ranging from 1.36 to 2.44 Å). However, its basic flavonol structure (rings A, B, and C) and a methyl group contributed to creating hydrophobic interactions with various amino acids, including Tyr63, His103, Ala143, Leu162, Phe163, Phe203, His224, Phe227, and Met228 (ranging from 4.04 to 5.44 Å). Consequently, it showed the highest binding affinity among query compounds in this group.

#### 3.9.3. Sulfonylurea Receptor (SUR)

**Figure 11:**
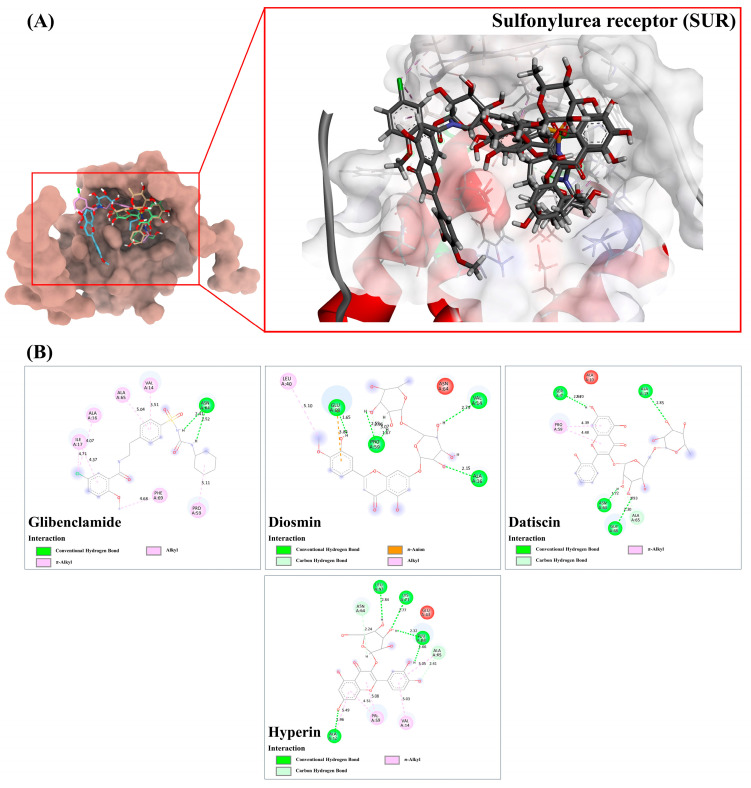
(**A**,**B**) showed 3D and 2D representations of the interaction of glibenclamide, diosmin, datiscin, and hyperin with sulphonylurea receptor (SUR).

In the sulfonylurea receptor (SUR), diosmin, datiscin, and hyperin displayed slightly stronger and comparable binding energies to glibenclamide, an anti-diabetic drug utilized to promote insulin secretion [24]. Glibenclamide, with a binding score of 50.76, formed two hydrogen bond interactions with Asn64 (2.41 and 2.52 Å) and exhibited seven hydrophobic interactions (alkyl and π-alkyl) with bond lengths ranging from 3.51 to 5.11 Å. In contrast, diosmin was projected to enhance insulin secretion more effectively by establishing five promising hydrogen bond interactions with Val14 (2.79 Å), Ala16 (2.15 Å), Pro59 (1.69 and 2.55 Å), and Glu68 (1.65 Å). Additionally, it engaged in two C-H interactions (2.97 and 3.07 Å) via the same proline residue and exhibited π-anion and alkyl-alkyl interactions at bond lengths of 3.84 and 5.10 Å, respectively. Notably, datiscin formed four robust hydrogen bonds (2.30–2.94 Å) and its π systems (rings A and C) could also create two promising π-alkyl bonds with Pro59 (4.39–4.48 Å), surpassing the reference drug in hydrogen bond formation. Similarly, hyperin, positioned well in the glibenclamide recognition site, established seven robust hydrogen bonds (1.66 to 2.84 Å) and five π-alkyl interactions (4.51–5.49 Å) with various active residues in the agonist-binding domain (Figure 11), potentially contributing to reduced blood glucose levels in the treated mice models.

#### 3.9.4. Glucagon Like Peptide-1 (GLP-1)

Metformin has been approved by the US Food and Drug Administration (FDA) as a glucagon-like peptide-1 (GLP-1) agonist for the management of Type 2 diabetes (T2D). It has been found to aid in reducing blood glucose levels [48]. Among the 21 docked metabolites, 16 displayed stronger binding activity than metformin, indicating potential agonistic effects on the peptide receptor. Metformin, with a binding energy of 37.25, formed three hydrogen bonds with Tyr42 and Gln45 and two π-cationic interactions with the same tyrosine residue within the GLP-1 receptor. Among the top four binders in the agonist binding site, guaijaverin, rutin, afzelin, and quercitrin were found to possess the highest binding scores (ranging from 61.30 to 62.25). Guaijaverin formed four robust hydrogen bonds with Asp53, Trp72, Asn82, and Ser84 (1.70–2.83 Å), while its quercetin core structure facilitated promising hydrophobic interactions—π-alkyl and amide-π stacked—within the drug-binding pocket (3.47–5.79 Å). The remaining three metabolites shared similar binding patterns, except that their glycoside moieties also participated in hydrogen bond formation with the active amino residues of the target peptide. For example, rutin utilized both aglycone and sugar moieties to form six robust hydrogen bonds with the active residues- Gln45, Asp53, Asn82, and Ser84 (1.66–2.93 Å), while the eight promising hydrophobic interactions (3.55–4.67 Å) were also presented.

**Figure 12:**
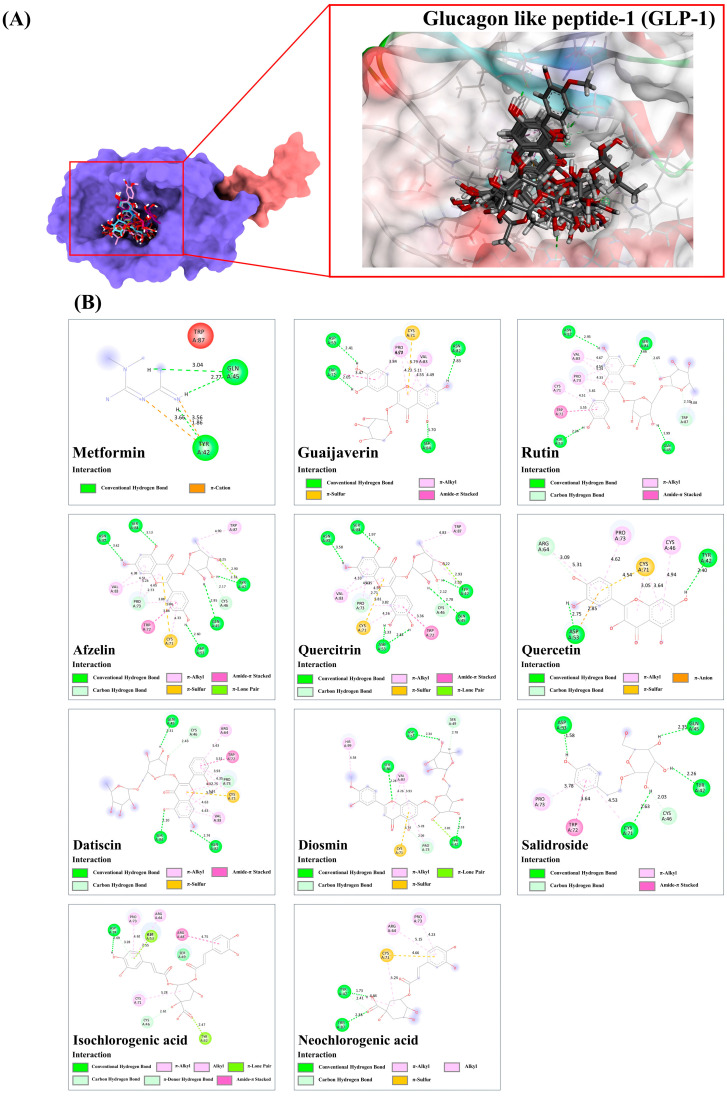
(**A**,**B**) showed 3D and 2D representations of the interaction of metformin, guaijaverin, rutin, afzelin, quercitrin, quercetin, diosmin, datiscin, salidroside, isochlorogenic acid, and neochlorogenic acid with glucagon-like peptide-1 (GLP1).

A similar pattern was observed in afzelin and quercitrin, which both demonstrated the establishment of π-lone pair and π-sulfur interactions within the GLP-1 structure. Afzelin, for instance, formed multiple hydrophobic interactions, in addition to four hydrogen bonds with Asp53, Pro73, Ser82, and Asn84, with bond lengths ranging from 2.63 to 3.13 Å. Its rhamnosyl group contributed three strong hydrogen bonds with lengths ranging from 2.17 to 2.85 Å. It is suggested that hydrophobic interactions enhanced the interaction of GLP-1 with most of the remaining phytochemicals, resulting in improved agonistic activity compared to the reference drug. As examples, quercetin, datiscin, and diosmin, with binding energies ranging from 54.77 to 55.22, were also considered. While quercetin established only two hydrogen bonds with Tyr42 and Asp53 (2.40–2.75 Å), it effectively formed hydrophobic interactions (π-anion, π-sulfur, and π-alkyl) with active residues, including Asp53 (2.85 Å), Arg64 (5.31 Å), Pro73 (4.62 Å), Cys71 (3.03–4.54 Å), and Cys46 (4.94 Å) via its basic ring structure. Similarly, datiscin and diosmin displayed similar binding patterns, featuring numerous hydrophobic interactions in the target region. The study revealed that three strong hydrogen bonds were formed initially with Gln45, Asn82, and Ser84, and π-anion, π-sulfur, and π-alkyl interactions were also generated with several active residues ranging from 3.31 to 5.43 Å. The subsequent interactions involved the basic C ring system and sugar moiety forming hydrogen bonds with Ser84, Asp53, and Tyr42 (2.18–2.34 Å), while six promising nonpolar interactions with Tyr42 (2.81 Å), Pro73 (5.28 Å), Cys71 (5.74 Å), Val83 (3.93–4.26 Å), and His99 (4.58 Å) stabilized the compound. Non-flavonoids, such as salidroside, isochlorogenic acid C, and neochlorogenic acid, were predicted to exert agonist activity due to higher binding energies and proper structural alignment in the target region relative to metformin. Salidroside established five solid hydrogen bonds with Asp53, Gln45, Tyr42, Cys46, and Cys71 (1.58–2.53 Å) and three significant hydrophobic interactions (3.78–4.53 Å) through its tyrosine-derived moiety. Isochlorogenic acid C and neochlorogenic acid shared similar binding features towards the GLP-1 receptor. With the aid of a caffeoyl-derived moiety, the former generated a strong hydrogen bond (Asp74: 2.69 Å) and a C-H bond (2.61 Å), while along with several hydrophobic interactions, including alkyl-alkyl (4.62–5.48 Å), π-alkyl (4.62–5.16 Å), π-lone pair (2.47–2.55 Å), π-donor hydrogen bond (2.61 Å), and amide-π stacked (4.75 Å) interactions within the drug interaction cavity. In neochlorogenic acid, the quinic acid-derived moiety not only formed three strong hydrogen bonds (1.73–2.41 Å) but also engaged in π-alkyl and alkyl-alkyl interactions with Tyr42 (4.84 Å) and Cys71 (5.24 Å). The caffeoyl moiety also established a π-sulfur interaction with Cys71 (4.66 Å) and two π-alkyl bonds (4.23–5.15 Å) within the same ligand-binding pocket (Figure 12).

#### 3.9.5. Insulin-like Growth Factor 1 Kinase (IGF1R)

**Figure 13:**
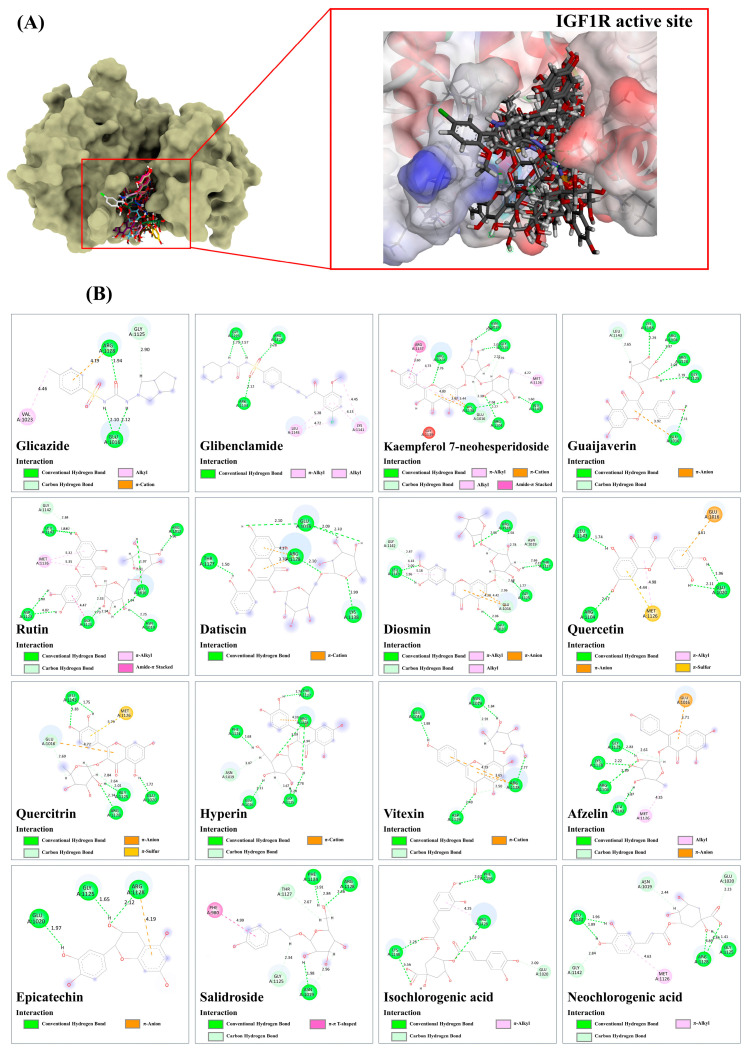
(**A**,**B**) showed 3D and 2D representations of the interaction of glicazide, glibenclamide, kaempferol 7-neohesperidoside, guaijaverin, rutin, afzelin, quercitrin, quercetin, diosmin, datiscin, hyperin, vitexin, epicatechin, salidroside, isochlorogenic acid, and neochlorogenic acid with insulin-like growth factor 1 kinase (IGF1R).

According to the docking results, numerous ligands exhibit superior binding affinity for IGF1R compared to gliclazide and glibenclamide, with respective binding scores of 45.93 and 61.03. Structural alignment reveals that all docked ligands are efficiently positioned within the same active region, indicating the results’ reliability. Gliclazide, a first-generation sulfonylurea, establishes four robust hydrogen bond interactions with Glu1016, Gly1225 (C-H), and Arg1128 (1.94–2.90 Å), along with a π-cationic bond with Arg1128 (4.19 Å) and an alkyl-alkyl interaction with Val1023 (4.46 Å) within the target receptor. Glibenclamide, a second-generation sulfonylurea drug, forms more effective hydrogen bonds with Arg1104 (2.12 Å), Arg1125 (1.75–2.57 Å), and Arg1128 (2.28 Å), as well as numerous hydrophobic interactions (4.15–5.28 Å) in the putative IGF1R active site. Kaempferol 7-neohesperidoside, guaijaverin, rutin, datiscin, and diosmin exhibit stronger binding activity than glibenclamide, suggesting their potential agonistic properties. Kaempferol 7-neohesperidoside ultimately formed nine hydrogen bonds with active residues, including Arg1128, Arg1019, Gly1125, Leu1143, Arg1104, Glu1016, and Lys1138 (with bond distances ranging from 1.60 to 2.76 Å), and participates in various types of hydrophobic interactions (ranging from 3.60 to 4.80 Å) within the drug recognition site. Similarly, diosmin, with a binding score of 67.66, establishes twelve hydrogen bond interactions with key amino acid residues (1.77–2.96 Å). Its flavone ring system (A, B, and C) engaged in π-anion, π-alkyl, and alkyl interactions with Leu1143 (4.14–5.18 Å) and Glu1016 (4.28–4.42) within the binding pocket. Remarkably, rutin exhibited numerous hydrogen-bond interactions with Leu1143 (1.63–2.42 Å), Gly1142 (2.84 Å), Glu1016 (1.64, 1.97, and 2.91 Å), Asn1019 (2.25 Å), Arg1012 (1.96 Å), Gly1125 (1.73, 2.33, and 2.94 Å), and Asp1123 (2.80 and 4.82 Å), while establishing amide-π stacked and π-alkyl interactions (4.47–5.32 Å) using the quercetin moiety. Similarly, guaijaverin and datiscin exhibited molecular recognition similar to glibenclamide, forming robust conventional hydrogen bonds with the two arginine residues (1125 and 1128). Consequently, they all showed identical binding energies to the target protein. Among the 14 metabolites, 9 had better binding energies than the first-generation drug, gliclazide, with fitness scores ranging from 47.14 to 60.11. Although glycosylation’s effect on flavonoid binding is complex and influenced by several factors, many glycosylated derivatives were more effective due to additional hydrogen bonding with target receptors. For instance, quercetin established four hydrogen bonds with Arg1104, Leu1143, and Glu1020 (1.74–2.17 Å) and demonstrated three promising hydrophobic interactions (4.44–4.98 Å) within the active region. Its glycosylated derivatives, quercitrin and hyperin, exhibited stronger binding activity due to a substantial number of hydrogen bond interactions with the IGF1R structure. The former established eight robust hydrogen bonds with various active residues: Glu1016 (2.69 Å), Leu1143 (1.73–1.83 Å), Arg1128 (2.14 Å), and Gly1125 (2.01, 2.64, 2.84 Å), while also interacting with Met1126 and Glu1016 through π-anion and π-sulfur interactions (4.72–5.79 Å). Based on the hydroxyl groups of the galactosyl moiety, the latter can form hydrogen bonds with Glu1016 (2.11 Å), Asn1019 (3.07 Å), Phe1124 (1.68 Å), Gly1125 (1.67, 1.78, and 2.28 Å), and Arg1128 (1.94 and 3.09 Å), engaging in π-cation interactions and hydrogen bonds through the aglycone core structure. The same reasoning may apply to quercetin-biosynthetically related structures, such as vitexin and afzelin. Glycosylation of their aglycone core structures, namely apigenin and kaempferol, resulted in enhanced binding energies relative to the referent drug. For vitexin, both the apigenin and glucosyl moieties instantaneously established six strong hydrogen bond residues (1.99–2.91 Å) with the drug-targeted region, while two promising π-cation interactions with the Arg1128 (3.65–4.19 Å) were also deduced in the target region. In the case of afzelin, the rhamnosyl moiety played an exclusive role in forming five robust hydrogen bond interactions with the putative residues, including Gly1125 (2.61–2.83 Å), Lys1138 (2.22 Å), Arg1104 (2.89 Å), and Leu1143 (1.87 Å), while also contributing two promising hydrophobic interactions (3.71–4.35 Å) through the methyl group (-CH_3_) and kaempferol moiety. Despite lacking glycosylation, epicatechin bound to the IGFR-1 structure via three hydrogen bonds (1.65–2.12 Å) and a π-cationic interaction (4.19 Å), rendering it more effective than gliclazide, possibly due to shorter hydrogen bond interactions (Figure 13).

Non-flavonoid natural products (salidroside, iso-, and neochlorogenic acids) showed stronger agonist activity than the reference drug owing to a wide variety of bonding interactions with the protein structure. Salidroside pronouncedly utilized its glucose-derived moiety to generate seven hydrogen bond interactions with Arg1128, Phe1124, Thr1127, Asn1019, and Gly1125, ranging between 1.91–2.96 Å, along with a π-π-T shaped interaction (4.99 Å), facilitated by the aromatic ring system of Phe980. Because of their isomeric properties, isochlorogenic acid and neochlorogenic acid exhibited similar binding features toward the target receptor; in particular, the caffeoyl-derived moiety was responsible for forming hydrophobic interactions (π-π-T shaped and π-alkyl). The initial one generated four H-bond interactions with Arg1128 (3.07 Å), Phe1159 (2.67 Å), and Lys1138 (2.25–5.56 Å), while a π-alkyl (Arg1228; 4.25 Å) also detected in the target region. The latter one also exhibited better hydrogen bonding capacity than the reference drug, forming eight hydrogen bonds with Gly1142 (2.84 Å), Leu1143 (1.89–1.96 Å), Asn1019 (2.44 Å), Glu1020 (2.23 Å), Gly1125 (1.41 Å), and Arg1128 (4.39–4.89 Å) within the putative binding pocket (Figure 13).

#### 3.9.6. Peroxisome Proliferator-Activated Receptor-Gamma (PPAR-γ)

**Figure 14:**
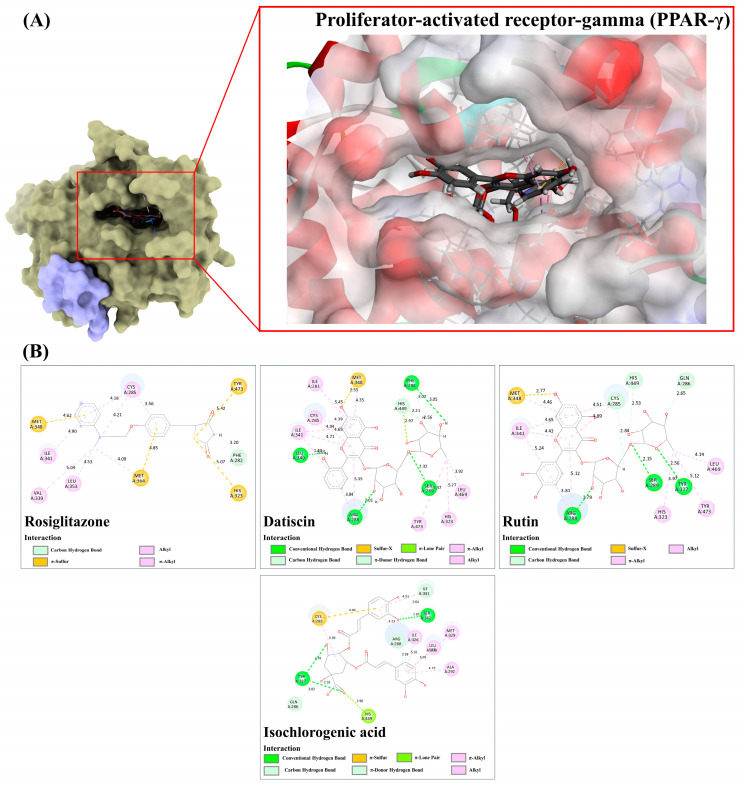
(**A**,**B**) showed 3D and 2D representations of the interaction of rosiglitazone, datiscin, rutin, and isochlorogenic acid with peroxisome proliferator-activated receptor-gamma (PPAR-γ).

Among the docked metabolites, rutin, datiscin, and isochlorogenic acid C were the three natural products that exhibited better binding affinity compared to the reference ligand (rosiglitazone), which had a fitness score of 72.76. The reference drug relied heavily on several hydrophobic interactions (π-sulfur, alkyl, and π-alkyl) ranging from 3.56 to 5.42 Å to effectively engage with the PPAR-γ receptor, with only one π-donor hydrogen bond (3.20 Å) detected. Conversely, rutin, with a binding score of 77.94, generated hydrogen-bond interactions with Arg288, Ser289, Tyr327, Cys285, His449, and Gln286 (1.79–2.84 Å) using its glycine moiety. It also formed numerous hydrophobic interactions with His323, Tyr473, Leu469, Arg288, Ile341, and Met348 (2.77–5.42 Å) at the drug recognition site via its quercetin ring system and the methyl group on the sugar moiety. A similar binding pattern was observed in the datiscin-PPAR-γ complex structure (Figure 14). Notably, the putative isochlorogenic acid C, with a fitness score of 72.48, reveals three promising H-bonds with Tyr327 (2.16–2.96 Å) and Ser342 (1.81 Å), and two C-H interactions (2.64–3.02 Å). Additionally, it sustains numerous hydrophobic interactions, including π-sulfur (4.84 Å), π-lone pair (2.90 Å), π-alkyl (4.51–5.16 Å), alkyl-alkyl (3.39 Å), and π-hydrogen bond (2.58 Å) with the expected region. This suggests that flavonoids with high structural complexity, particularly those of two sugar moieties, have significant potential as PPAR-γ activators, aligning with our wet lab experiments.

#### 3.9.7. Glycogen Phosphorylase-1 (GP-1)

**Figure 15:**
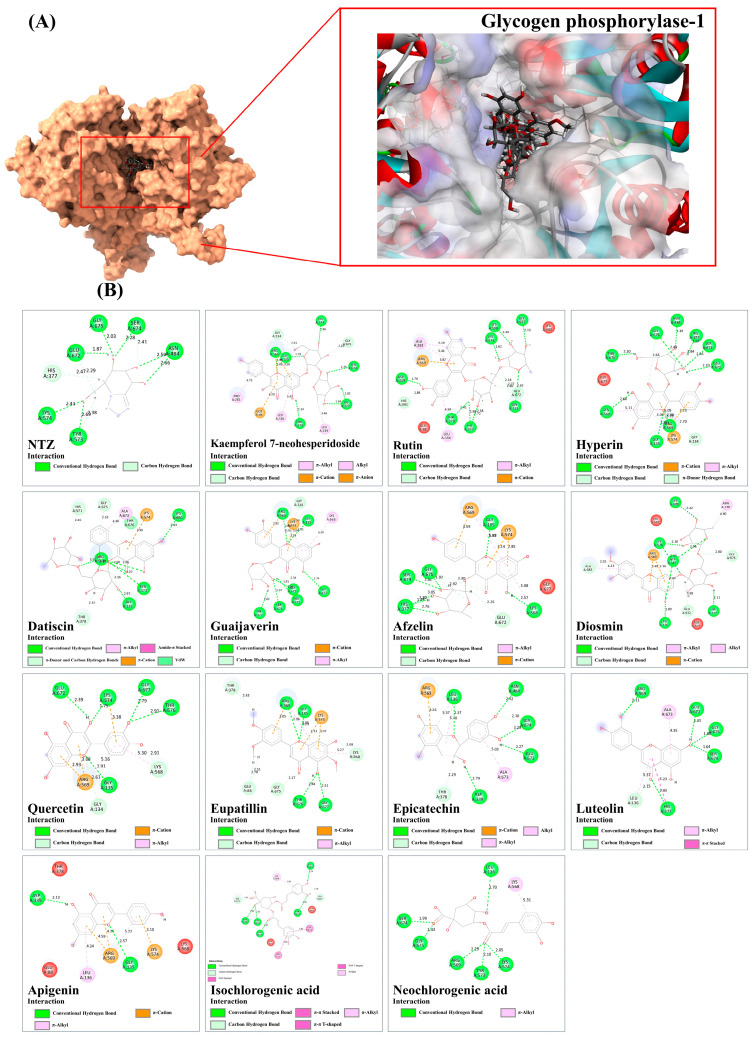
(**A**,**B**) showed 3D and 2D representation of the interaction of nojirimycin tetrazole (NTZ), kaempferol 7-neohesperidoside, guaijaverin, rutin, afzelin, eupatillin, quercetin, diosmin, datiscin, hyperin, luteolin, epicatechin, apigenin, isochlorogenic acid, and neochlorogenic acid with glycogen phosphorylase-1 (GP1).

We studied the structure of glycogen phosphorylase complexed with nojirimycin tetrazole (NTZ) to investigate whether metabolites from PK could compete with GP1 at the drug recognition site, potentially lowering blood glucose levels by decreasing hepatic glucose production [49]. Out of the 21 docked metabolites, 18 displayed higher fitness scores than NTZ against the glycogen-degrading enzyme. Among these, 18 metabolites surpassed NTZ’s energy level of 39.75. Flavonoids with one to two sugar moieties exhibited stronger binding compared to simple phenolic compounds. NTZ formed six hydrogen bonds with active residues, including His377, Glu672, Ser674, Ala673, Gly135, and Thr676, ranging from 1.70 to 2.69 Å. Kaempferol 7-neohesperidoside revealed multiple hydrogen bonds with Arg649 (2.71 Å), Arg569 (1.91–1.98 Å), Thr573 (3.05 Å), His377 (2.20–2.68 Å), Gly135 (2.62 Å), Glu672 (2.75 Å), Thr676 (2.59–2.90 Å), Gly677 (2.63–2.68 Å), and Val567 (2.92 Å), while also forming hydrophobic interactions with adjacent residues (2.79–5.38 Å), which were well-localized in the specific binding region. Like Kaempferol, other glycosidic flavonoids, including rutin, hyperin, datiscin, guaijaverin, afzelin, and diosmin, effectively interacted with substrate/drug recognition residues (Gly135, Gly675, Glu672, Arg569, and Ala673), forming multiple hydrogen bonds and electrostatic and hydrophobic interactions. For instance, hyperin formed fourteen hydrogen bonds with the catalytic residues through the combination of galactosyl and quercetin moieties, spanning between 1.97 and 3.05 Å. Concurrently, the flavonol ring system facilitated π-cation and π-alkyl interactions, lending greater stability and specificity to the drug recognition pocket. Although quercetin was expected to exhibit more potent biological activity than the reference drug, its derivatives, rutin and guaijaverin, showed stronger binding energy due to the presence of glycone moieties. The initial compound established eleven promising hydrogen bond interactions (1.42–2.98 Å), which were approximately 2.75 times greater than those found in the GP1/quercetin complex structure. The subsequent compound, with a binding score of 70.53, formed ten robust hydrogen bonds with active amino acid residues, including His377, Ser674, Glu672, Gly675, Thr676, Gly677, Gly135, and Tyr573, with bond lengths ranging from 1.82 to 2.96 Å. Moreover, it engaged in π-alkyl and π-cation interactions (2.92–5.35 Å) through the π-systems of the flavonol moiety (Figure 14). Non-glycosylated flavonoids, including eupatillin, epicatechin, luteolin, and apigenin exhibited higher binding energies than the NTZ drug. Among these flavonoids, eupatillin, for example, formed eight strong hydrogen bonds with key active residues, ranging from 1.69 to 3.10 Å, representing a twofold increase compared to the reference drug. For non-flavonoid substances, most, except for quinic acid, vanillic acid, and shikimic acid, exhibited a strong binding affinity with the GP1 structure compared to the drug, suggesting potential antidiabetic properties. Regarding the two chlorogenic acid derivatives, isochlorogenic acid C, with a binding affinity of 80.66, can generate seven H-bonds with the active amino acids Ser674, Gly675, Ala673, Thr676, Val562, and His377, with bond lengths between 1.14 and 2.99 Å. Meanwhile, the two benzene rings of the caffeoyl-derived moieties were responsible for forming hydrophobic interactions with His571, Tyr573, and Lys568, ranging between 3.83 and 5.99 Å. A similar binding pattern was found for neochlorogenic acid, except this compound created only one hydrophobic interaction with Lys568 (5.31 Å) occurring at the caffeic acid-derived portion (Figure 15).

### 3.10. LC-MS/MS-Based Phytochemical Profiling of the Plu kaow Ethanolic Extract with Structural Annotation Using MetFrag Web Service and Sirius Tandem with CSI:FingerID and CANOPUS

High-resolution UPLC-ESI-QTOF-MS/MS in negative ion mode was employed to verify the presence of phenolic compounds in PK extracts, such as quercetin, quercitrin, and chlorogenic acids, due to its superior robustness and sensitivity compared to positive ion mode [50]. As expected, flavonoids and phenolic acids were the primary phytochemicals in the PK, with hit scores from the MS^2^ spectra ranging from 63.3% to 100% and a mass error between 6.18 and 48.03 ppm (Table 5). The MetFrag web service was utilized to verify their correspondence by comparing it against appropriate databases. Twenty-one out of 22 query subjects could be annotated, with fourteen metabolites defined as the first-ranked structure among other potential structures postulated. For instance, the ion with *m*/*z* 607.1352 [M-H]^−^, which has a matching score of only 63.3% and an error of about −51.221552 ppm, was best annotated as diosmin (1st) with all mass peaks (3/3) explained, indicating increased reliability in the prediction. A similar trend was observed in the two negative precursor ions with *m*/*z* 289.0851 and 593.1339, where the mass errors exceeded the accepted criteria (>20 ppm) [51]. According to the NORMAN database, the initial compound was determined to be epicatechin, with a score of 1.0 and 30/37 peaks explained. Utilizing the same database, the second compound was most effectively annotated as kaempferol-7-O-neohesperidroside, with an F-score of 1.0 and 3 out of 4 mass peaks explained. Additionally, the negative ions with *m*/*z* values 191.0593, 353.0955, 299.1150, 289.0851, 607.1352, 431.10015, 463.0958, 433.0804, 447.1004, 147.0460, 285.0419, 301.0373, 269.0475, and 343.0839 matched perfectly with those in the MS library, ranking as the top candidate (1st). On the other hand, the remaining metabolites were annotated among the top ten candidates (e.g., 2nd, 3rd, and 7th), likely due to the software’s inability to differentiate isomeric structures and its limited database. For instance, *m*/*z* 431.1025 (RT: 5.07 min) was identified as vitexin (3rd), with a matching score and peaks explained that matched those of the top two candidates (1st and 2nd) in the NORMAN databases. The putative isochlorogenic acid C (*m*/*z* 515.1216) ranked seventh among 168 candidates in the PubChem database. However, searching biological databases did not annotate the query subject. Similarly, the *m*/*z* 593.1549 [M-H]^−^ was not explained, even after searching against all suspected databases. Therefore, the annotation accuracy of the metabolites needs validation by other tools to strengthen the results.

As detailed in Table 5, the identification accuracy was greatly strengthened after submitting the same raw mass dataset to Sirius with the CSI:FingerID web service and CANOPUS. Notably, most metabolites were annotated as the top-ranked candidate (1st) among the hundred structures retrieved, as their InChIKey and InChI codes were in the CSI:FingerID negative mode data set (accessed on 17 April 2024 at https://www.csi-fingerid.uni-jena.de/v2.6/api/fingerid/trainingstructures?predictor=2), suggesting these results should be highly dependable. For instance, the putative datiscin (*m*/*z* 593.1549 [M-H]^−^), which MetFrag did not identify, was re-ranked as the 5th top candidate, implying it may be more elucidative. Likewise, the query negative ions at *m*/*z* 167.0362 (6th), 173.0450 (4th), 609.1544 (2nd), 431.1025 (3rd), 515.1216 (5th), and 343.089 (7th) were entirely reassigned to the top candidate (1st) in both molecular formula and structural annotations after comparison with the suspected databases. Meanwhile, annotating the putative mass peaks corresponding to kaempferol-7-O-neohesperidroside (*m*/*z* 593.1339), diosmin (*m*/*z* 607.1352), and neochlorogenic acid (*m*/*z* 353.0955) requires reliance on those well-explained by MetFrag and other characterizations, presumably due to their absence in the software’s training set. CANOPUS revealed that they were simple phenolics, flavonoids, and non-phenolic substances, as detailed below.

#### 3.10.1. Simple Phenolics

**Figure 16:**
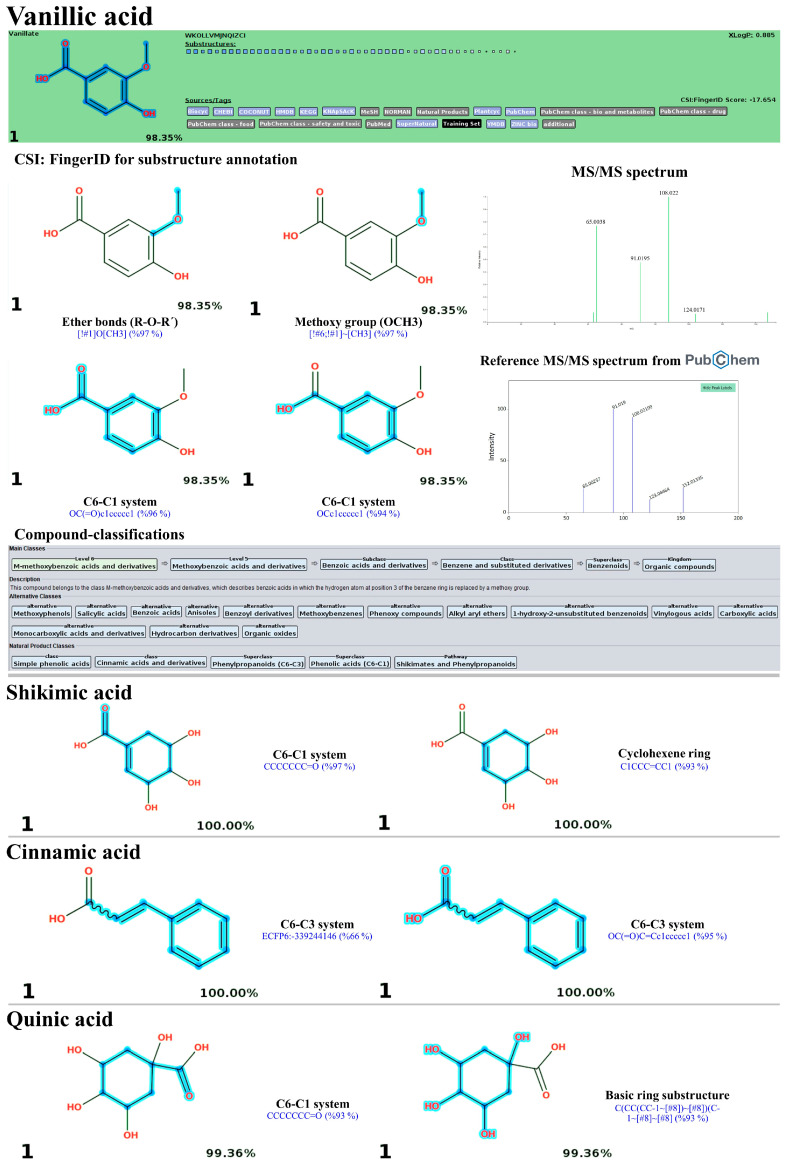
Structural annotation of simple phenolics detected from the plu kaow ethanolic extract.

Compound classification suggested four query metabolites derived from the shikimate and phenylpropanoid pathways. The high-resolution isotope pattern analysis revealed their correct neutral formula, with the identity scores ranging from 99.63 to 100%. CSI:FingerID consistently confirmed that they were quinic acid, vanillic acid, cinnamic acid, and shikimic acid, characterized as the top candidate (1st) among a hundred structures retrieved from various databases (e.g., Plantcyc, Natural Products, and COCONUT). Several molecular fingerprints were detected that illustrated the basic core structures (C_6_-C_1_ and C_6_-C_3_), carboxylic acid, hydroxy, and methoxy groups. For instance, the mass peak at *m*/*z* 167.0362 [M-H]^−^, corresponding to vanillic acid, exclusively contained substructures indicating the presence of a methoxy group (−OCH_3_) and ether bonds (R–O–R’) within the benzoic ring system (C_6_-C_1_), such as ‘[!#1]O[CH3] (97%)’ and ‘[!#6;!#1]~[CH3] (97%)’. This is partly supported by the detection of product ions at *m*/*z* 124.0171, 108.022, 91.0195, and 65.0038, indicating the successive losses of a carboxyl group, a methyl group, and two fragmented ions from the phenolic ring system. Karonen and Pihlava have also detected similar fragmented ions [52]. Since the four queried metabolites share the same biosynthetic routes, the successful annotation of the remaining metabolites—shikimic acid, quinic acid, and cinnamic acid—might also be strengthened by presenting substructures representing their basic ring structures, such as ‘CCCCCC=O (C_6_-C_1_; 97%; shikimic acid)’, ‘OC(=O)C=Cc1ccccc1(C_6_-C_3_; 95%; cinnamic acid)’ and ‘CCCCCC=O (C_6_-C_1_; 93%; quinic acid)’ (Figure 16).

#### 3.10.2. Flavonoids and Glycoside Analogues

**Figure 17:**
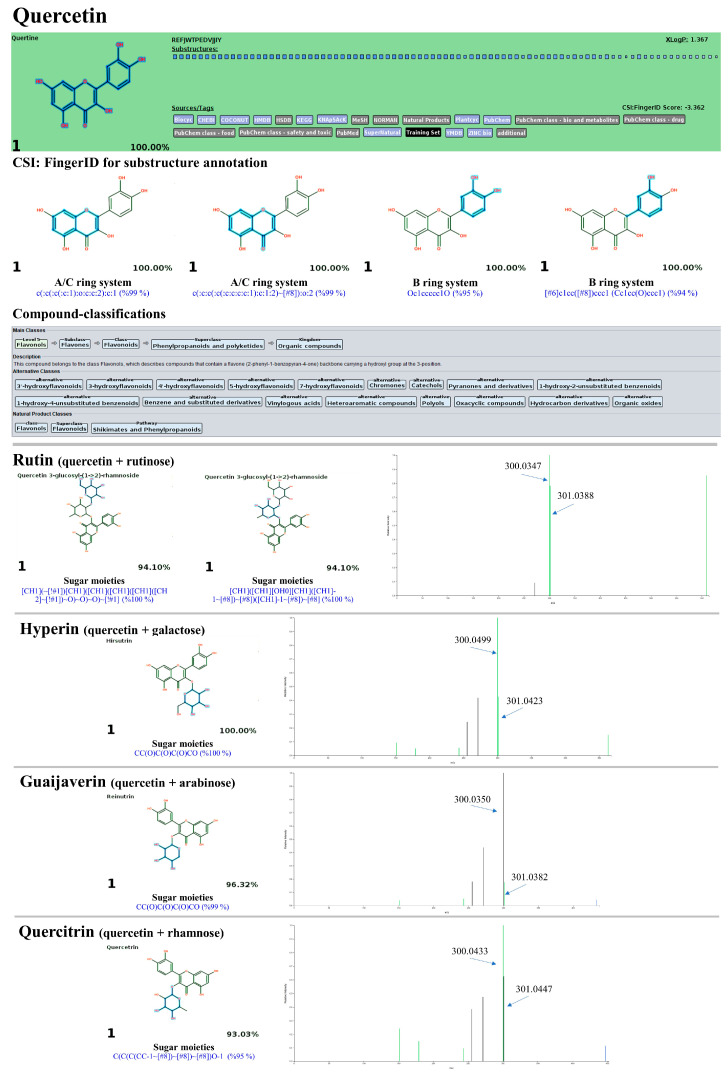
Structural annotation of quercetin and its four glycosylated derivatives.

Shikimate and phenylpropanoid pathways were predicted to synthesize numerous flavonoids in the PK. Twelve out of fourteen query subjects were successfully annotated by Sirius (with CSI:FingerID and CANOPUS), giving rise to vital information about their formula, structure, and classification. Meanwhile, the structural annotation of putative kaempferol-7-O-neohesperidoside (*m*/*z* 593.1339) and diosmin (*m*/*z* 607.1494) relies on predictions by MetFrag, as both were identified as the top-ranked candidates. Quercetin and its derivatives (rutin, hyperin, guaijaverin, and quercitrin) shared numerous fingerprints of the C_6_(ring A)-C_3_(ring C)–C_6_(ring B) structure but differed in their sugar moieties. For instance, substructures encoded as ‘c(:c(:c(:c:c:1):o:c:c:2):c:1 (99%)’ and ‘c(:c:c(:c(:c:c:c:c:1):c:1:2)~[#8]):o:2’ received posterior probability scores of 99%. This indicates the interconnection between the A and C rings within the quercetin structure (Figure 16). The B ring systems (*p*-coumaroyl-CoA derived moiety) encoded by ‘Oc1ccccc1O (95%)’ and ‘[#6]c1cc([#8]ccc1) (Cc1cc(O)ccc1) (94%)’ were also detected within the same candidate. On the contrary, substructures indicating the presence of glycoside moieties, including glucose coupled with rhamnose (rutin), galactose (hyperin), arabinose (guaijaverin), and rhamnose (quercitrin), were found solely in the four quercetin derivatives, encoded by ‘[CH1](~[!#1])[CH1]([CH1]([CH1]([CH1]([CH2]~[!#1] with [CH1](CH1][OH0][CH1]([CH1]-1~[#8]) ~[#8])(CH1]-1~[#8])~[#8] (100%; glucosylrhanoside)’, ‘CC(O)C(O)C(O)CO (100%; galactose)’, ‘CC(O)C(O)C(O)CO (99%; arabinose)’, and ‘C(C(C(CC-1~[#8])~[#8])~[#8])O-1 (95%; glucose)’, respectively (Figure 17). Notably, the successive loss of sugar moieties between the C-ring and the glycosidic linkage, leading to the fragment at *m*/*z* 301 (quercetin), was also observed in these four glycosylated products’ MS/MS spectrum.

**Figure 18:**
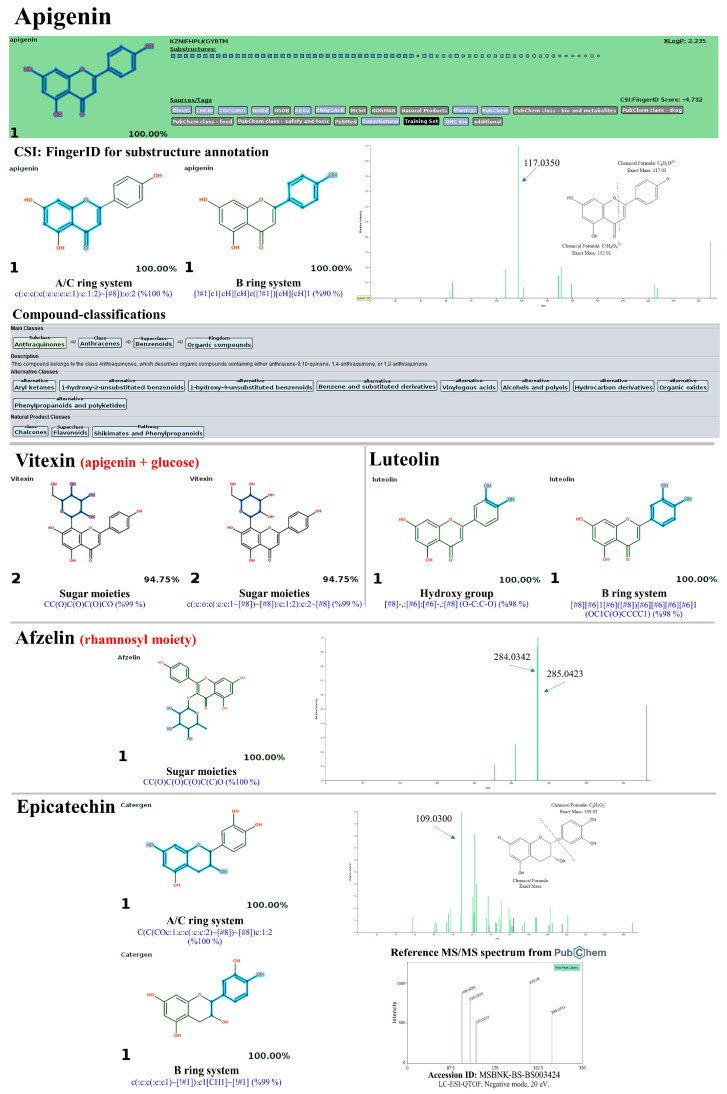
Structural annotation of apigenin, vitexin, luteolin, afzelin, and epicatechin.

Similarly, vitexin (*m*/*z* 431.1001) was distinguished from its parent apigenin by detecting sugar fingerprints encoded by ‘CC(O)C(O)C(O)CO (99%)’ and ‘ c(:c:o:c(:c:c(:c:c:1~[#8])~[#8]):c:1:2):c:2~[#8] (99%)’, with the glucosyl loss in the ESI-MS/MS spectrum, yielding the fragmented ion at *m*/*z* 269.0482 (apigenin). The putative afzelin (*m*/*z* 431.10015 [M-H]^−^), the top-ranked candidate (1st), possesses numerous substructures indicating glycosidic moiety ‘CC(O)C(O)C(O)C(C)O’ with the posterior probability score of 100% (Figure 18). In the MS/MS spectrum, the product ion with *m*/*z* 285.0423 (base peak) indicated the complete loss of the rhamnosyl unit from the aglycone structure (kaempferol), thus supporting the annotation of this query subject. Putative luteolin (*m*/*z* 285.0419) could also be distinguished from its biosynthetic precursor, apigenin, apparently due to the clear detection of two chemical codes for the B-ring system—‘[#8]-,:[#6]:[#6]-,[#8] (O-C:C-O) (98%)’ and ‘[#8][#6]1[#6]([#8])[#6][#6][#6][#6]1 (OC1C(O)CCCC1)’, and vice versa for the apigenin structure. Its MS/MS spectrum also included fragment ions at *m*/*z* 151.0044 and *m*/*z* 133.0302 (base peak), representing a common fragmentation pathway used to characterize the substitution patterns on luteolin’s A and B rings. The successive loss of the ring B system, indicated by the ion at *m*/*z* 109.03 (base peak), was observed in the putative epicatechin (*m*/*z* 289.0851; Rt = 4.18 min), which ranked as the top candidate for both formula and structural annotations.

**Figure 19:**
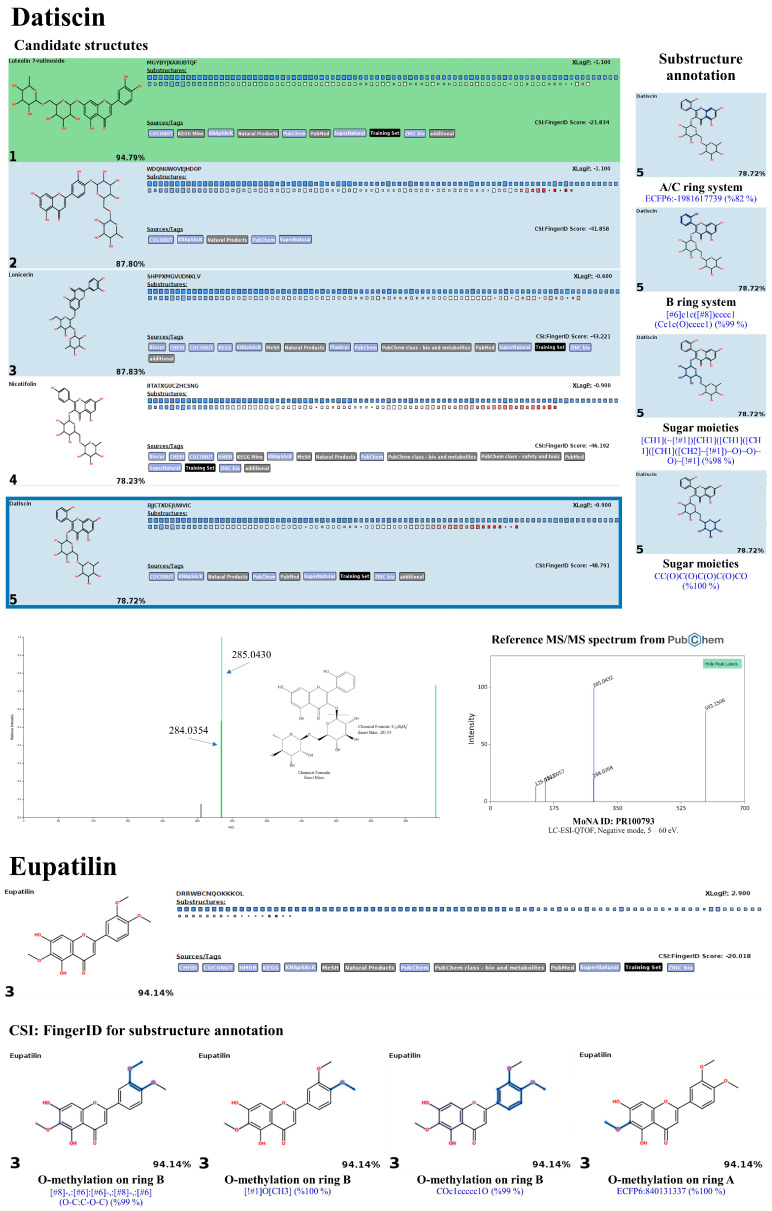
Structural annotation of datiscin and eupatilin.

Datiscin (*m*/*z* 593.1549) was identified as the best-hit candidate (5th) among a hundred compounds retrieved from the CSI:FingerID web service. However, it might be possible to consider the remaining candidates—luteolin 7-rutinoside (1st), lonicerin (2nd), nicotifolin (3rd), and nicotifolin (4th)—as unsuitable structures since the software used still have limitations in distinguishing between the trained isomeric substances. This metabolite showed a fragmentation profile similar to authentic datiscin, tentatively characterized by a fragment ion at *m*/*z* 285 (base peak), indicating the loss of the sugar moiety from datiscetin (core structure) (accessed on 17 April 2024 at https://pubchem.ncbi.nlm.nih.gov/compound/Datiscin#section=LC-MS&fullscreen=true), while substructures indicating aglycone and sugar moieties were postulated by the CSI:FingerID web service. Hence, they were anticipated to be the same substance. A similar phenomenon was observed with eupatillin (*m*/*z* 343.0839; Rt = 8.42 min), which ranked as the top candidate (3^rd^/100^th^), narrowing the search to biological databases (KEGG and HMDB) significantly elevated its ranking to second and first candidates, respectively. This was further supported by the detection of substructures indicating successive O-methylations on the basic flavone system (rings A and B), which are denoted by ‘[#8]-,:[#6]-,:[#6]-,:[#8]-,:[#6] (O-C:C-O-C) (99%)’, ‘[!#1]O[CH3] (100%)’, ‘Coc1ccccc1O (99%)’, and ‘ECFP6: 840,131,337 (100%)’ (Figure 19).

#### 3.10.3. Non-Flavonoid Substances

**Figure 20:**
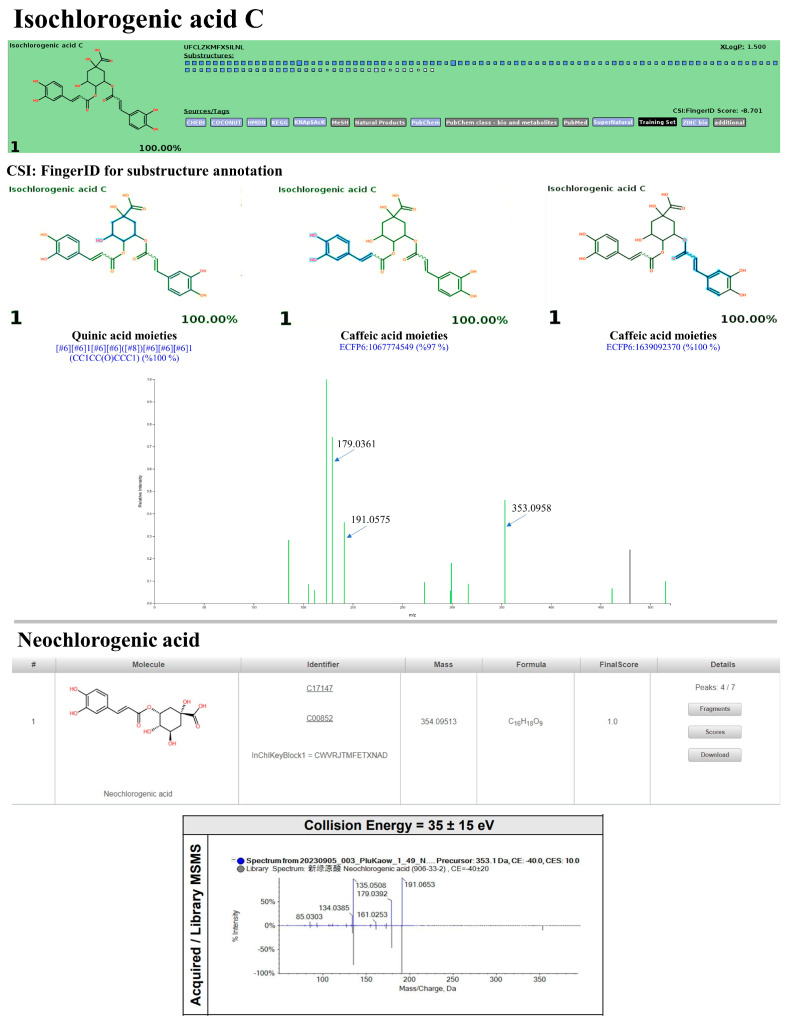

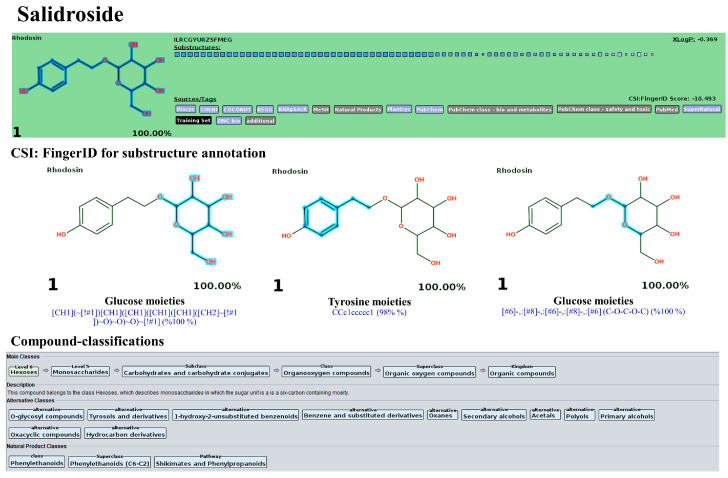
Structural annotation of isochlorogenic acid C, neochlorogenic acid, and salidroside using MetFrag, library matching, and Sirius (CSI;FingerID + CANOPUS).

With Sirius, the rank of putative isochlorogenic acid C (*m*/*z* 515.1216) was shifted to the best-hit candidate (1st) compared to its previous ranking with MetFrag (7th/165th). Substructure detection revealed a quinic acid di-esterified with two caffeic acids present in this candidate. For example, one coded by ‘[#6][#6]1[#6][#6]([#8])[#6][#6][#6]1 (CC1CC(O)CCC1) (100%)’ signifying the connection of carboxylic and trihydroxy groups with the six-membered ring of quinic acid. The esterified caffeic acids, deduced by ‘ECFP6:1639092370 (100%)’ and ‘ECFP6: 1067774549 (97%)’, were also detected (Figure 20). Its MS/MS spectrum also showed product ions at *m*/*z* 179 and 191, indicating the presence of caffeic acid and quinic acid moieties.

Neochlorogenic acid, which has an *m*/*z* of 353.0955, is thought to be another type of chlorogenic acid present in the PK. While it was not ranked as coherently as it was in the referent library and MetFrag (1st), it is possible that the accuracy of CSI:FingerID (as part of Sirius) decreased by approximately 27.6% when the pre-existing MS/MS training is removed [53]. This issue is underscored by the fact that neochlorogenic acid (InChIKey= CWVRJTMFETXNAD-NXLLHMKUSA-N) was not included in the structure training set (accessed on 17 April 2024 at <https://www.csi-fingerid.uni-jena.de/v2.6/api/fingerid/trainingstructures?predictor=2>), which resulted in poor identification of the query metabolite. Consequently, it may also be feasible to rule out 3-O-caffeoylquinic acid (1st) and 4-caffeoylquinic acid (2nd) as biased structures, as both compounds have already been trained and exhibited lower fit scores (81.3% and 84.4%, respectively) compared to neochlorogenic acid (97.7%) when re-checked against the natural products HR-MS/MS library (v2.0) and NIST 2017 MS/MS library. Salidroside was tentatively identified with its molecular ion (*m*/*z* 299.1150) and ranked as the top candidate (1st/100th). Molecular fingerprint detections have verified the presence of tyrosine-derived portions (e.g., “CCc1ccccc1 (98%)”) and glucose moiety (e.g., “[CH1](~[!#1])[CH1]([CH1]([CH1](CH1]([CH2]~[!#1])~O)~O)~O)~[!#1] (100%)”), confirming the biosynthetic constituents of this natural product.

## 4. Discussion

The present study was conducted on experimental diabetic rats to investigate the effects of plu kaow ethanolic extract (PK) on hyperglycemia and hyperlipidemia, along with its potential molecular mechanism. Streptozotocin (STZ) is a compound that is commonly used to induce diabetes in laboratory animals. It enters the β-cells of islets of Langerhans through a glucose transporter (GLUT2) and causes damage to the DNA, leading to necrosis. This ultimately results in decreased insulin levels and the development of hyperglycemia (Figure 1), which are both key characteristics of diabetes mellitus [34,54]. Additionally, STZ induces a proinflammatory state, a lipidemic state, and a redox imbalance, all of which are also associated with diabetes [24].

Our research demonstrated that administering PK orally to diabetic rats resulted in a substantial decrease in their blood glucose levels and a noticeable improvement in their weight loss (as shown in Figure 2 and Figure 4). This favorable outcome could be attributed to PK’s ability to regenerate pancreatic β-cells and release insulin or its capacity to stimulate insulin release from β-cells by inhibiting ATP-sensitive K^+^ channels [55]. This action is similar to that of conventional hypoglycemic drugs (glibenclamide), which stimulate insulin release and restrict glucagon secretion from the pancreas [24]. To better understand these phenomena, we have considered several well-established pathways and antidiabetic receptors, including α-amylase, α-glucosidase, sulfonylurea receptor (SUR), glycogen phosphorylase (GP), glucagon-like peptide-1 (GLP-1), insulin-like growth factor 1 (IGF1R) kinase, and peroxisome proliferator-activated receptor-gamma (PPAR-γ) [31]. Our study’s in-silico analysis showed that the query ligands were predicted to exhibit antidiabetic activity by targeting multiple receptors (as detailed in Table 4). Among the 21 docked metabolites, 18 exhibited higher fitness scores than nojirimycin tetrazole (NTZ), while rutin and datiscin displayed better binding affinity than the standard drug rosiglitazone (72.76). Additionally, kaempferol 7-neohesperidoside, guaijaverin, rutin, datiscin, and diosmin revealed a stronger binding affinity for IGF1R compared to glibenclamide (61.03) and gliclazide (45.93), demonstrating their potential agonistic properties, as discussed in the results section.

Metformin, with a binding energy of 37.25 (expressed as the GoldScore function), functions as a glucagon-like peptide-1 (GLP-1) and reduces blood glucose levels [48]. This study also indicated that among the docked metabolites, 16 exhibited stronger binding activity than metformin, suggesting potential agonistic effects on the peptide receptor. Among the top four binders in the agonist binding site, guaijaverin, rutin, afzelin, and quercitrin were identified as possessing the highest binding scores (ranging from 61.30 to 62.25).

The compounds kaempferol 7-neohesperidoside, isochlorogenic acid, rutin, datiscin, and diosmin have demonstrated promising activity, as they exhibit stronger binding energy compared to the reference drug acarbose, which achieved a fitness score of 66.44. Acarbose, a glycemic control agent, established nine hydrogen bond interactions with various active residues, including Tyr151, Arg195, Lys200, Glu240, and Asp300, with bond lengths ranging between 1.65 and 2.97 Å in the α-amylase structure. However, in the sulfonylurea receptor (SUR), diosmin, datiscin, and hyperin exhibited slightly stronger and comparable binding energies to glibenclamide. These phytochemicals improve the ability of sulfonylureas to interact with receptors on pancreatic beta cells, blocking ATP-sensitive potassium channels, subsequently leading to the opening of calcium channels and the subsequent production of insulin [56]. 

Recent reports have indicated that PK possesses several active compounds that may contribute to its potential benefits for diabetes and lipid regulation [12]. These compounds consist of flavonoids, volatile oils, and other substances such as gallic acid, quercitrin, quercetin-3-O-beta-D-galactopyranoside, houttuynoside A, houttuynamide A, rutin, resveratrol, decanoyl acetaldehyde, and chlorogenic acid [22]. Our molecular docking studies have confirmed these findings. Moreover, our study has revealed the presence of quinic acid, D-pyroglutamic acid, shikimic acid, galactinol, neochlorogenic acid, vanillic acid, N-acetyl-L-phenylalanine, salidroide, epicatechin, salicylcurcumin, isochlorogenic acid C, diosmin, rutin, vitexin, afzelin, hyperin, datiscin, guaiaverin, quercetin, quercitrin, abscisic acid, kaempferol-7-O-neohesperidoside, cinnamic acid, luteolin, apigenin, and eupatilin in the ethanolic extract of PK.

Previous studies have demonstrated that quinic acid activates Ca^2+^-dependent mitochondrial function and enhances insulin secretion from beta cells [55]. Meanwhile, vanillic acid has been found to suppress the overexpression of inflammatory mediators such as NF-κB, TNF-α, and COX-2 and also promotes the up-regulation of nuclear factor-erythroid 2-related factor 2 (Nrf-2) proteins, which play a crucial role in diabetes and diabetic nephropathy [57]. Additionally, cinnamic acid and its derivatives are known to have insulin secretagogue effects. The dual activity of cinnamic acid in insulin signaling and secretion reveals its role in activating insulin-mediated glucose transport through the involvement of GLUT4 and a PI3-K-independent pathway [58]. Furthermore, literature reviews have shown that newly isolated compounds also found in PK improve blood glucose levels by inhibiting α-amylase and α-glucosidase activity, regulating sulfonylurea receptor (SUR), glycogen phosphorylase (GP), GLP-1, insulin-like growth factor 1 (IGF1R) kinase, and peroxisome proliferator-activated receptor-gamma (PPAR-γ) (Table 6) [59,60,61,62,63,64,65,66,67,68,69,70,71,72,73,74,75,76,77,78,79,80]. Our research further confirmed PK’s potential in treating diabetes through histopathological findings. The diabetic groups showed shrinkage, necrosis, and damaged β-cell populations. However, upon administering PK, these effects were reversed (Figure 7A1–A5). Our findings are consistent with previous studies, which have demonstrated that PK possesses excellent hypoglycemic properties [81], improves insulin resistance, alleviates endotoxemia by modulating the gut microbiota, and enhances the effectiveness of metformin when used in combination [12].

Dyslipidemia is a common complication in diabetic rats, characterized by elevated serum TG, TC, and LDL-C and decreased HDL-C. Insulin plays a vital role in regulating carbohydrate and lipid metabolism in the body. It helps to inhibit the activity of hormone-sensitive lipases found in fat cells, which reduces the release of free fatty acids into the bloodstream [82]. In normal conditions, insulin helps to increase the receptor-mediated removal of LDL cholesterol and activates lipoprotein lipase, which breaks down triglycerides (TG). This process helps to prevent hypercholesterolemia, atherosclerosis, and related cardiovascular complications [34,83]. However, in diabetic conditions, the activity of hormone-sensitive lipases increases, causing the breakdown of lipids from peripheral deposits, and more free fatty acids to be released into the bloodstream [84]. Based on this study, PK significantly (*p* < 0.05) reduced triglycerides, total cholesterol, and LDL-cholesterol levels while increasing HDL-cholesterol levels (Table 1). This could potentially alleviate dyslipidemia, inhibit lipid synthesis, and further improve diabetes symptoms. The active compounds found in plu kaow extract, including shikimic acid, vanillic acid, quinic acid, rutin, diosmin, and kaempferol, have been shown to activate phosphorylation of AMP-activated protein kinase (AMPK)/acetyl-coenzyme A carboxylase (ACC) and reduce the expression of MID1 Interacting Protein 1 (MID1IP1). According to Kim et al. (2019), this suggests that plu kaow may have a significant hypolipogenic effect [85]. A recent in vitro study provides evidence that PK inhibits lipid accumulation induced by high glucose in human HepG2 hepatocytes by inducing adenosine monophosphate-activated protein kinase (AMPK) signaling [86].

The liver plays a vital role in maintaining blood glucose levels by carrying out glycogenolysis and gluconeogenesis, especially in the post-absorptive state. In diabetes, high blood sugar levels cause the kidneys to reabsorb excess glucose, resulting in damage to the liver and kidneys. Consequently, specific markers such as SGPT, SGOT, alkaline phosphatase, and serum creatinine levels may increase. Alam et al. (2014) reported that quercetin supplementation significantly improves these markers [87]. This study confirmed that administration of PK to diabetic rats decreased serum enzyme activity in comparison to the untreated diabetic group (Table 2 and Figure 3) since PK contains quercetin and other potential phytonutrients like polyphenols and flavonoids [23]. However, the imbalance of nitrogen in diabetes can result in a decrease in protein synthesis, which in turn can cause weight loss and an increase in serum creatinine levels, indicating kidney damage. Moreover, the levels of serum creatinine were significantly (*p* < 0.05) reduced after treatment with PK compared with the untreated diabetic group (Figure 3), which suggests that the plant prevents kidney damage and associated functional impairment. In addition, histological examinations of diabetic liver and kidneys revealed damaged hepatocytes and renal cells. After treatment with PK, the histoarchitectural alterations were significantly improved (Figure 7B1–B5,C1–C5), demonstrating its healing and rejuvenating potential. As stated by Arya et al. (2014), quercetin and quinic acid inhibit the expression of the pro-apoptotic protein Bax and notably enhance the expression of the anti-apoptotic protein Bcl-2 in kidney tissues. Consequently, this leads to the regeneration of damaged cells and plays a defensive role in the kidneys of diabetic rats [88].

This study also addressed the consequences of cardiac markers, particularly creatine kinase, which holds clinical significance as an indicator for diagnosing myocardial and skeletal muscle disorders [89]. In diabetic rats, elevated levels of both creatine kinase (CK) and creatine kinase MB (CKMB) were observed (Figure 3). However, after treatment with PK, a trend towards lower levels of creatine kinase and creatine kinase MB was observed. The hypothesis is that the bioactive compound of PK activates the sulphonylurea (SU) receptor, which may override any harmful effects of inhibition of the K_ATP_ channels and prevent ATP ischemic preconditioning. A similar scenario was observed in glibenclamide-treated rats. According to Loubani et al. (2005), the sulphonylurea (SU) receptor 1 (pancreatic) has two binding sites, a benzamido site and a sulphonylurea site, whereas the sulphonylurea (SU) receptor 2 (cardiac) has only the benzamido binding site. Glibenclamide has both moieties, whereas other hypoglycemic drugs like gliclazide have only the sulphonylurea moiety, and thus, glibenclamide does not block the protective effect of activation of PKC or p38MAPK/JNK [90]. Further studies are required to elucidate the mechanism of these changes.

A decline in body weight was evident in our study of diabetic rats, which was due to the loss of structural proteins and muscle mass [24]. However, the PK-treated rats gained significant (*p* < 0.01) weight at the end of the treatment compared to their initial weight (Figure 4). The possible mechanism behind the PK extract’s effectiveness is linked to an increase in insulin secretion, which in turn helps regulate blood sugar levels and prevents further weight loss. The relative organ weights (organ/body weight) of the experimental rats were also considered in this study. It was found that the administration of PK or glibenclamide did not significantly (*p* < 0.05) affect the organ weights of the rats in comparison to the corresponding control rats, as shown in Figure 6B. 

The results displayed in Figure 4 indicate a substantial (*p* < 0.05) rise in food consumption among the groups treated with PK compared to their respective control groups. The mechanism by which PK affects food intake could involve the involvement of AMP-activated protein kinase (AMPK) in the acetyl-CoA (ACC)-malonylCoA pathway in the hypothalamus [91,92]. Previous research has shown that PK and glibenclamide increase the expression of GLUT4 and the signaling pathways such as the AMPK [93,94]. The active components found in PK extract, including rutin, shikimic acid, vanillic acid, quinic acid, diosmin, kaempferol, guaijaverin, afzelin, and quercitrin, may also play a significant role in AMPK regulation.

Based on the findings, it can be concluded that the bioactive secondary metabolites present in PK have the potential to treat diabetes and dyslipidemia through various mechanisms, such as increasing insulin secretion, inhibiting intestinal alpha-glucosidase, and activating glucokinase. Additionally, these compounds can improve liver glycogen levels and lower serum cholesterol and LDL concentrations, which is similar to the effects of the standard diabetic drug, glibenclamide. Therefore, PK could be a promising candidate for the management and treatment of diabetes and dyslipidemia in the future. However, additional systematic research is necessary to assess its efficacy in both preclinical and clinical trials.

## 5. Conclusions

In conclusion, the findings of this research imply that the plu kaow ethanolic extract (PK) comprises numerous phytocompounds. Molecular docking investigations indicate that kaempferol 7-neohesperidoside, isochlorogenic acid, guaijaverin, rutin, datiscin, and diosmin are the most promising compounds for their hypoglycemic effects on various antidiabetic targets, such as α-amylase, α-glucosidase, sulfonylurea receptor (SUR), glycogen phosphorylase (GP), glucagon-like peptide-1 (GLP-1), insulin-like growth factor 1 (IGF1R) kinase, and peroxisome proliferator-activated receptor-gamma (PPAR-γ). Moreover, rutin and datiscin have the potential to activate PPAR-γ. The PK extract also exhibited potential effects on hyperglycemia and improved biochemical parameters related to dyslipidemia, liver, kidney, and heart function. However, further research is needed, particularly longer-duration studies on chronic models, to elucidate the exact mechanism of action of the extract and its components and develop it as a potent antidiabetic and antihyperlipidemic drug.

## Figures and Tables

**Table 1:** Effect of plu kaow ethanolic extract (500 mg/kg/day) on lipid profile (*n* = 8).

	Healthy Control			Diabetes			
	HC	HPK	*p*	DC	DD	DPK	*p*
Total Cholesterol (mg/dL)	89.5 ± 9.1	94.7 ± 13.5	ns	154.0 ± 6.9	82.6 ± 5.6 ***	89.4 ± 5.9 ***	<0.001
Triglyceride (mg/dL)	81.5 ± 11.7	59.1 ± 4.1	ns	171.3 ± 10.7	41.4 ± 11.0 ***	52.4 ± 11.0 ***	<0.001
HDL (mg/dL)	38.3 ± 9.5	60.0 ± 12.4	ns	24.2 ± 2.7	45.4 ± 2.7 ***	43.9 ± 2.5 ***	<0.001
LDL (mg/dL)	39.4 ± 3.3	29.7 ± 4.8	ns	103.1 ± 10.3	29.9 ± 5.4 ***	35.2 ± 5.2 ***	<0.001
VLDL (mg/dL)	12.9 ± 2.2	9.2 ± 1.9	ns	32.6 ± 2.7	8.4 ± 2.4 ***	10.3 ± 2.2 ***	<0.001
TC/HDL ratio	3.1 ± 0.5	1.6 ± 0.14	<0.05	7.4 ± 1.3	2.0 ± 0.3 ***	2.3 ± 0.3 ***	<0.001
LDL/HDL ratio	1.7 ± 0.4	0.62 ± 0.15	ns	4.6 ± 0.6	0.74 ± 0.18 ***	0.90 ± 0.18 ***	<0.001

Values are expressed as mean ± SD, (*n* = 8). HC (Healthy control); HPK (Healthy treated group); DC (Diabetic control); DPK (Diabetic treated group); DD (Diabetic drug group). Output from student T test or one-way ANOVA and *p* value indicates significant difference between two groups. For healthy control group HC was considered reference and for diabetic groups, DC was considered reference group. *** *p* < 0.001, ns = non-significant.

**Table 2:** Evaluation of the effect of plu kaow ethanolic extract on hepatic enzyme activity.

	Healthy Control			Diabetes			
	HC	HPK	*p*	DC	DD	DPK	*p*
SGPT (U/L)	78.2 ± 9.4	93.5 ± 12.3	ns	106.4 ± 19.2	47.7 ± 8.8 **	77.8 ± 12.9 *	*
SGOT (U/L)	115.1 ± 17.1	86.8 ± 23.8	ns	144.3 ± 27.8	29.8 ± 7.1 ***	69.6 ± 18.6 *	*
AST/ALT Ratio	1.74 ± 0.27	1.39 ± 0.45	ns	1.5 ± 0.29	0.85 ± 0.23	1.0 ± 0.23	ns
ALP (U/L)	193.6 ± 16.4	283.9 ± 24.9	ns	480.9 ± 117.0	255.6 ± 44.9	340.9 ± 36.4	ns

Values are expressed as mean ± SD, (*n* = 8). HC (Healthy control); HPK (Healthy treated group); DC (Diabetic control); DPK (Diabetic treated group); DD (Diabetic drug group). Output from student T test or one-way ANOVA and *p* value indicates significant difference between two groups. For healthy control group HC was considered reference and for diabetic groups, DC was considered reference group. * *p* < 0.05, ** *p* < 0.01, *** *p* < 0.001, ns = non-significant.

**Table 3:** Effect of plu kaow ethanolic extract on antioxidant activity.

Sample	Total Phenolic Content	ABTS	DPPH
	(GAE)/g	IC_50_ (mg/mL)	
Plu kaow ethanolic extract	84.87	0.44	0.16
Gallic acid	-	0.03	0.003
Quercetin	-	0.04	0.018

**Table 4:** Molecular docking illustrating the target specificity of polyphenols detected from the plu kaow ethanolic extract against various proteins related to the hyperglycemia condition.

Compound	PubChem (CID)	Binding Score/RMSD (Å)						
		α-Amylase/1.5606 Å *	α-Glucosidase/1.8760 Å **	SUR/0.8158 Å *	GLP-1/1.3217 Å *	GP/0.7958 Å *	IGF1R/0.2632 Å ***	PPAR-γ/0.6223 Å *
Cinnamic acid	44539	35.49	19.50	27.33	33.28	35.31	35.17	35.14
Kaempferol 7-neohesperidoside	5483905	67.74	42.87	46.65	54.59	66.83	67.50	54.63
Luteolin	5280455	60.65	38.05	41.15	54.25	52.27	47.14	54.12
Epicatechin	72276	55.31	35.60	36.37	55.20	52.43	51.66	57.95
Quercetin	5280343	62.41	39.36	37.98	55.22	54.64	51.49	53.62
Quercitrin	5280459	58.79	42.89	45.05	62.91	65.78	54.08	62.83
Salidroside	159278	54.12	37.22	38.32	53.71	54.73	55.50	51.81
Vanillic acid	8468	39.42	21.48	31.64	32.01	38.06	37.20	38.77
Neochlorogenic acid	5280633	53.04	40.97	40.38	53.99	59.52	53.71	63.04
Vitexin	5280441	54.15	45.04	44.75	50.85	60.50	51.67	70.74
Quinic acid	6508	44.20	25.17	24.86	30.32	39.60	41.99	40.01
Isochlorogenic acid C	5315832	69.77	38.06	38.36	54.92	55.98	57.23	60.76
Guaijaverin	5481224	55.19	44.91	45.23	62.65	73.78	62.01	65.34
Rutin	5280805	70.95	50.75	48.85	61.30	59.71	66.67	77.94
Afzelin	5316673	59.14	41.19	44.18	61.91	63.93	53.45	62.40
Hyperin	133568467	62.06	45.35	50.10	58.82	77.11	60.11	55.13
Datiscin	10054207	71.30	44.79	53.97	54.77	68.60	61.36	73.93
Shikimic acid	8742	39.01	24.59	25.84	31.69	34.82	39.21	38.12
Diosmin	5281613	75.28	50.43	50.99	55.08	84.12	67.66	67.96
Apigenin	5280443	55.86	37.62	35.90	53.47	51.83	44.83	51.25
Eupatilin	5273755	61.45	38.53	37.26	49.05	55.86	43.68	63.09
Rosiglitazone	77999	-	-	-	-	-	-	72.76
Acarbose	9811704	66.44	49.78	-	-	-	-	-
Luteolin ^†^	5280455			37.37	-	-	-	-
Glibencamide (Glyburide)	3488	-	-	50.76	-	-	61.03	-
Metformin	4091	-			37.25	-	-	-
Nojirimycin tetrazole (NTZ)	41684	-	-	-	-	45.32	-	-
Glicazide	3475	-	-	-	-		45.93	-

* The binding scores were expressed as “GoldScore function” ** The binding scores were expressed as “ASP function” *** The fitness scores were expressed as “ChemPLP function” ^†^ According to Van et al. (2022) [31].

**Table 5:** Molecular formula and rank annotation of selected metabolites primarily detected in the plu kaow ethanolic extract using UPLC-ESI(-)-QTOF-MS/MS coupled with Sirius (v.5.8.6) and MetFrag web service.

No.	UPLC-ESI (-)-QTOF-MS/MS								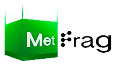	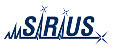
	Retention Time (min)	Metabolite	Predicted Neutral Formula (Sirius Score %) *	Neutral mass ***	Theoretical (*m*/*z*) [M-H]^− †^	Exp. Mass (*m*/*z*)	Mass Error (ppm) ^‡^	Library Score (%) ^#^	Rank/DB (F1 Score)	Rank/DB Training (YES/NO) ^§^
1	0.76	Quinic acid	C_7_H_12_O_6_ (99%)	192.06	191.0556	191.0593	19.58	89.8	1st/KEGG (1.0)	1st/All Databases (YES)
2	0.65	Shikimic acid	C_7_H_10_O_5_ (100%)	174.05	173.0450	173.0450	0	100	4th/NORMAN (0.7462)	1st/All Databases (YES)
3	1.88	Neochlorogenic acid	C_16_H_18_O_9_ (100%)	354.10	353.0873	353.0955	23.34	97.7	1st/NORMAN (1.0)	ND. (NO)
4	3.06	Vanillic acid	C_8_H_8_O_4_ (99.63%)	168.04	167.0344	167.0362	10.57	91.7	6th/KEGG (0.7846)	1st/All Databases (YES)
5	3.78	Salidroside	C_14_H_20_O_7_ (97.17%)	300.12	299.1131	299.1150	6.42	98.8	1st/KEGG (1.0)	1st/All Databases (YES)
6	4.18	Epicatechin	C1_5_H_14_O_6_ (100%)	290.08	289.0712	289.0851	48.03	90.6	1st/NORMAN (1.0)	1st/All Databases (YES)
7	4.85	Isochlorogenic acid C	C_25_H_24_O_12_ (90.93%)	516.13	515.1190	515.1216	5.13	86.5	7th/PubChem (0.9858)	1st/All Databases (YES)
8	4.90	Diosmin	C_28_H_32_O_15_ (N.D) **	608.17	607.1663	607.1352	−51.22	63.3	1st/KEGG (1.0)	ND. (NO)
9	5.01	Rutin	C_27_H_30_O_16_ (100%)	610.15	609.1455	609.1544	14.61	96.0	2nd/NORMAN (0.0)	1st/All Databases (YES)
10	5.07	Vitexin	C_21_H_20_O_10_ (100%)	432.11	431.0987	431.1025	10.84	96.3	3th/NORMAN (1.0)	1st/All Databases (YES)
11	5.85	Afzelin	C_21_H_20_O_10_ (94.77%)	432.11	431.0987	431.10015	5.39	95.2	1st/KEGG (1.0)	1st/All Databases (YES)
12	5.18	Hyperin	C_21_H_20_O_12_ (100%)	464.10	463.0877	463.0958	17.59	99.5	1st/KEGG (1.0)	1st/All Databases (YES)
13	5.29	Datiscin	C_27_H_30_O_15_ (79.27%)	594.16	593.1507	593.1549	7.17	99.4	ND.	5th/All Databases (YES)
14	5.40	Guaijaverin	C_20_H_18_O_11_ (100%)	434.08	433.0771	433.0804	7.64	95.6	1st/NORMAN (1.0)	1st/All Databases (YES)
15	5.57	Quercitrin	C_21_H_20_O_11_ (100%)	448.10	447.0927	447.1004	17.13	99.5	1st/NORMAN (1.0)	1st/All Databases (YES)
16	6.35	Kaempferol-7-O-neohesperidroside	C_27_H_30_O_15_ (N.D.) **	594.16	593.1507	593.1339	−28.24	100	1st/NORMAN (1.0)	ND. (NO)
17	6.41	Cinnamic acid	C_9_H_8_O_2_ (99.95%)	148.05	147.0446	147.0460	9.49	95.3	1st/KEGG (1.0)	1st/All Databases (YES)
18	6.52	Luteolin	C_15_H_10_O_6_ (95.80%)	286.05	285.0399	285.0419	6.96	94.8	1st/KEGG (1.0)	1st/All Databases (YES)
19	6.58	Quercetin	C_15_H_10_O_7_ (92.71%)	302.04	301.0348	301.0373	8.21	97.5	1st/NORMAN (1.0)	1st/All Databases (YES)
20	7.08	Apigenin	C_15_H_10_O_5_ (98.89%)	270.05	269.0450	269.0475	9.29	99.3	1st/KEGGs (1.0)	1st/All Databases (YES)
21	8.42	Eupatilin	C_18_H_16_O_7_ (100%)	344.09	343.0818	343.0839	6.18	98.9	3rd/KEGG (0.988)	3rd/All Databases 2nd/KEGG 1st/HMDB

* Annotation is based on both high-resolution isotope pattern analysis (as defined by Sirius) and the MetFrag web service. ** Formula annotation is based on MetFrag web service. *** Neutral mass annotation is defined by MetFrag web service. ^†^ The theoretical mass/charge (*m*/*z*) values were calculated by a web service (accessed on 17 April 2024 at https://www.sisweb.com/referenc/tools/exactmass.htm). ^‡^ Calculated by a mass error calculation tool (accessed on 17 April 2024 at https://warwick.ac.uk/fac/sci/chemistry/research/barrow/barrowgroup/calculators/mass_errors/). ^§^ Tracking from the structural training set (accessed on 17 April 2024 at https://www.csi-fingerid.uni-jena.de/v2.6/api/fingerid/trainingstructures?predictor=2). ^#^ Matching against tandem mass spectral data was derived from the Natural Products HR-MS/MS Library (version 2.0) and NIST 2017 MS/MS library.

**Table 6:** Phytochemical compounds of plu kaow ethanolic extract to treat diabetes mellitus that act on various pathways and target proteins to increase insulin or insulin sensitivity or inhibit glucose release from food.

Mechanism of Action	Target Compounds of PK Containing Anti-Diabetic Properties	References
Inhibition of α-amylase activity	Kaempferol 7-neohesperidoside, isochlorogenic acid, rutin, datiscin, diosmin	[59,60,61,62,63,64]
Inhibition of α-glucosidase activity	Datiscin, diosmin, rutin, isochlorogenic acid	[59,60,61,62,63,64]
Regulation of sulfonylurea receptor (SUR)	Diosmin, datiscin, hyperin	[63,65,66]
Inhibition of Glycogen phosphorylase	Kaempferol, glycosidic flavonoids, rutin, hyperin, datiscin, guaijaverin, afzelin, and diosmin	[67,68,69,70]
Release of glucagon-like peptide-1 (GLP-1)	Guaijaverin, afzelin, rutin, quercitrin, salidroside, isochlorogenic acid C, neochlorogenic acid	[71,72,73,74]
Modulation of insulin-like growth factor 1 (IGF1R) kinase	Kaempferol 7-neohesperidoside, guaijaverin, rutin, datiscin, diosmin	[75,76,77]
Inhibition of Peroxisome proliferator-activated receptor-gamma (PPAR-γ)	Rutin, kaempferol, datiscin, diosmin	[78,79,80]

## Data Availability

The data that support the findings of this study are available from the corresponding author upon reasonable request. The data are not publicly available due to privacy and/or ethical restrictions.

## References

[1] Alam U., Asghar O., Azmi S., Malik R.A. (2014). General aspects of diabetes mellitus. Handb. Clin. Neurol..

[2] Abdul Basith Khan M., Hashim M.J., King J.K., Govender R.D., Mustafa H., Al Kaabi J. (2020). Epidemiology of type 2 diabetes—Global burden of disease and forecasted trends. J. Epidemiol. Glob. Health.

[3] Sun H., Saeedi P., Karuranga S., Pinkepank M., Ogurtsova K., Duncan B.B., Stein C., Basit A., Chan J.C., Mbanya J.C. (2022). IDF Diabetes Atlas: Global, regional and country-level diabetes prevalence estimates for 2021 and projections for 2045. Diabetes Res. Clin. Pract..

[4] Papatheodorou K., Banach M., Bekiari E., Rizzo M., Edmonds M. (2018). Complications of Diabetes 2017. J. Diabetes Res..

[5] Vergès B. (2015). Pathophysiology of diabetic dyslipidaemia: Where are we?. Diabetologia.

[6] Jialal I., Singh G. (2019). Management of diabetic dyslipidemia: An update. World J. Diabetes.

[7] Hirano T. (2014). Abnormal lipoprotein metabolism in diabetic nephropathy. Clin. Exp. Nephrol..

[8] Bonilha I., Zimetti F., Zanotti I., Papotti B., Sposito A.C. (2021). Dysfunctional high-density lipoproteins in type 2 diabetes mellitus: Molecular mechanisms and therapeutic implications. J. Clin. Med..

[9] Lorenzo C., Hartnett S., Hanley A.J., Rewers M.J., Wagenknecht L.E., Karter A.J., Haffner S.M. (2013). Impaired fasting glucose and impaired glucose tolerance have distinct lipoprotein and apolipoprotein changes: The insulin resistance atherosclerosis study. J. Clin. Endocrinol. Metab..

[10] Kashyap S.R., Osme A., Ilchenko S., Golizeh M., Lee K., Wang S., Bena J., Previs S.F., Smith J.D., Kasumov T. (2018). Glycation reduces the stability of ApoAI and increases HDL dysfunction in diet-controlled type 2 diabetes. J. Clin. Endocrinol. Metab..

[11] Nandini H.S., Naik P.R. (2019). Antidiabetic, antihyperlipidemic and antioxidant effect of Vincamine, in streptozotocin-induced diabetic rats. Eur. J. Pharmacol..

[12] Verma R.S., Joshi N., Padalia R.C., Singh V.R., Goswami P., Kumar A., Iqbal H., Verma R.K., Chanda D., Chauhan A. (2017). Chemical Composition and Allelopathic, Antibacterial, Antifungal, and Antiacetylcholinesterase Activity of Fish-mint (*Houttuynia cordata* Thunb.) from India. Chem. Biodivers..

[13] Luo Q., Meng P.H., Jiang D.W., Han Z.M., Wang Z.H., Tan G.F., Zhang J. (2022). Comprehensive assessment of *Houttuynia cordata* Thunb., an important medicinal plant and vegetable. Agronomy.

[14] Li A., Li S., Wu X., Lu H., Huang M., Gu R., Wei L., He A. (2015). Influence of light intensity on the yield and quality of *Houttuynia cordata*. Plant Prod. Sci..

[15] Fotev Y.V., Kukushkina T.A., Chankina O.V., Belousova V.P. (2018). Houttuynia (*Houttuynia cordata* thunb.)–new vegetable and medicinal crop for Russia (morphological features and biochemical composition). Veg. Crop. Russ..

[16] Dong X.Y., Yang L.P. (2017). Systematic analysis of components and contents in *Houttuynia cordata* Thunb. Tradit. Med. Res..

[17] Wu Z., Deng X., Hu Q., Xiao X., Jiang J., Ma X., Wu M. (2021). *Houttuynia cordata* Thunb: An ethnopharmacological review. Front. Pharmacol..

[18] Pan X., Li H., Chen D., Zheng J., Yin L., Zou J., Zhang Y., Deng K., Xiao M., Meng L. (2021). Comparison of Essential Oils of *Houttuynia cordata* Thunb. from Different Processing Methods and Harvest Seasons Based on GC-MS and Chemometric Analysis. Int. J. Anal. Chem..

[19] Chou S.C., Su C.R., Ku Y.C., Wu T.S. (2009). The constituents and their bioactivities of *Houttuynia cordata*. Chem. Pharm. Bull..

[20] Yang L., Jiang J.G. (2009). Bioactive components and functional properties of *Hottuynia cordata* and its applications. Pharm. Biol..

[21] Pradhan S., Rituparna S., Dehury H., Dhall M., Singh Y.D. (2023). Nutritional profile and pharmacological aspect of *Houttuynia cordata* Thunb. and their therapeutic applications. Pharmacol. Res. -Mod. Chin. Med..

[22] Kim J., Kim S.R., Choi Y.H., Shin J.Y., Kim C.D., Kang N.G., Park B.C., Lee S. (2020). Quercitrin stimulates hair growth with enhanced expression of growth factors via activation of MAPK/CREB signaling pathway. Molecules.

[23] Laldinsangi C. (2022). The therapeutic potential of *Houttuynia cordata*: A current review. Heliyon.

[24] Kahksha, Alam O., Al-Keridis L.A., Khan J., Naaz S., Alam A., Ashraf S.A., Alshammari N., Adnan M., Beg M.A. (2023). Evaluation of Antidiabetic Effect of Luteolin in STZ Induced Diabetic Rats: Molecular Docking, Molecular Dynamics, In Vitro and In Vivo Studies. J. Funct. Biomater..

[25] Wang H.Y., Bao J.L. (2012). Effect of *Houttuynia cordata* aetherolea on adiponectin and connective tissue growth factor in a rat model of diabetes mellitus. J. Tradit. Chin. Med..

[26] Ma Q., Wei R., Wang Z., Liu W., Sang Z., Li Y., Huang H. (2017). Bioactive alkaloids from the aerial parts of *Houttuynia cordata*. J. Ethnopharmacol..

[27] Kumar M., Prasad S.K., Hemalatha S. (2016). In vitro study on glucose utilization capacity of bioactive fractions of *Houttuynia cordata* in isolated rat hemidiaphragm and its major phytoconstituent. Adv. Pharmacol. Pharm. Sci..

[28] Folin O., Ciocalteu V. (1927). On tyrosine and tryptophane determinations in proteins. J. Biol. Chem..

[29] Klamrak A., Nabnueangsap J., Narkpuk J., Saengkun Y., Janpan P., Nopkuesuk N., Chaveerach A., Teeravechyan S., Rahman S.S., Dobutr T. (2023). Unveiling the Potent Antiviral and Antioxidant Activities of an Aqueous Extract from *Caesalpinia mimosoides* Lamk: Cheminformatics and Molecular Docking Approaches. Foods.

[30] Xiao F., Xu T., Lu B., Liu R. (2020). Guidelines for antioxidant assays for food components. Food Front..

[31] Van L.V., Pham E.C., Nguyen C.V., Duong N.T., Le Thi T.V., Truong T.N. (2022). In vitro and in vivo antidiabetic activity, isolation of flavonoids, and in silico molecular docking of stem extract of *Merremia tridentata* (L.). Biomed. Pharmacother..

[32] Alinezhad H., Azimi R., Zare M., Ebrahimzadeh M.A., Eslami S., Nabavi S.F., Nabavi S.M. (2013). Antioxidant and antihemolytic activities of ethanolic extract of flowers, leaves, and stems of *Hyssopus officinalis* L. Var. angustifolius. Int. J. Food Prop..

[33] Rahman S.S., Reja M.M., Islam M.R., Islam M.M., Rouf S.M., Rahman M.H. (2023). Proximate nutrient analysis of elephant apple (*Dillenia indica*) fruit and its hypoglycemic, and hypolipidemic potentials in alloxan-induced diabetic rats. Food Humanit..

[34] Latifi E., Mohammadpour A.A., Fathi B., Nourani H. (2019). Antidiabetic and antihyperlipidemic effects of ethanolic *Ferula assa-foetida* oleo-gum-resin extract in streptozotocin-induced diabetic wistar rats. Biomed. Pharmacother..

[35] Rahman S.S., Salauddin H.M., Rahman M., Muhsin M.M., Rouf S.M. (2021). Nutritional composition and antidiabetic effect of germinated endosperm (*Borassus flabellifer*), tuber (*Amorphophallus paeoniifolius*) and their combined impact on rats. Biochem. Biophys. Rep..

[36] Wu J., Yan L.J. (2015). Streptozotocin-induced type 1 diabetes in rodents as a model for studying mitochondrial mechanisms of diabetic β cell glucotoxicity. Diabetes Metab. Syndr. Obes. Targets Ther..

[37] Madhuri K., Naik P.R. (2017). Ameliorative effect of borneol, a natural bicyclic monoterpene against hyperglycemia, hyperlipidemia and oxidative stress in streptozotocin-induced diabetic Wistar rats. Biomed. Pharmacother..

[38] Ibrahim M., Parveen B., Zahiruddin S., Gautam G., Parveen R., Khan M.A., Gupta A., Ahmad S. (2022). Analysis of polyphenols in *Aegle marmelos* leaf and ameliorative efficacy against diabetic mice through restoration of antioxidant and anti-inflammatory status. J. Food Biochem..

[39] Kumar M., Prasad S.K., Krishnamurthy S., Hemalatha S. (2014). Antihyperglycemic Activity of *Houttuynia cordata* Thunb. in Streptozotocin-Induced Diabetic Rats. Adv. Pharmacol. Pharm. Sci..

[40] Shanak S., Saad B., Zaid H. (2019). Metabolic and epigenetic action mechanisms of antidiabetic medicinal plants. Evid. -Based Complement. Altern. Med..

[41] Date K., Satoh A., Iida K., Ogawa H. (2015). Pancreatic α-amylase controls glucose assimilation by duodenal retrieval through N-glycan-specific binding, endocytosis, and degradation. J. Biol. Chem..

[42] Donley V.R., Hiskett E.K., Kidder A.C., Schermerhorn T. (2005). ATP-sensitive potassium channel (K ATP channel) expression in the normal canine pancreas and in canine insulinomas. BMC Vet. Res..

[43] Sesti G., Sciacqua A., Cardellini M., Marini M.A., Maio R., Vatrano M., Succurro E., Lauro R., Federici M., Perticone F. (2005). Plasma concentration of IGF-I is independently associated with insulin sensitivity in subjects with different degrees of glucose tolerance. Diabetes Care.

[44] McCormack J.G., Westergaard N., Kristiansen M., Brand C.L., Lau J. (2001). Pharmacological approaches to inhibit endogenous glucose production as a means of anti-diabetic therapy. Curr. Pharm. Des..

[45] Garber A.J. (2011). Long-acting glucagon-like peptide 1 receptor agonists: A review of their efficacy and tolerability. Diabetes care.

[46] Kota B.P., Huang T.H., Roufogalis B.D. (2005). An overview on biological mechanisms of PPARs. Pharmacol. Res..

[47] Pokharkar O., Lakshmanan H., Zyryanov G.V., Tsurkan M.V. (2023). Antiviral potential of *Antillogorgia americana* and elisabethae natural products against nsp16–nsp10 complex, nsp13, and nsp14 proteins of sars-cov-2: An in silico investigation. Microbiol. Res..

[48] Bailey C.J. (2017). Metformin: Historical overview. Diabetologia.

[49] Agius L. (2008). Glucokinase and molecular aspects of liver glycogen metabolism. Biochem. J..

[50] Wolf S., Schmidt S., Müller-Hannemann M., Neumann S. (2010). In silico fragmentation for computer assisted identification of metabolite mass spectra. BMC Bioinform..

[51] Dührkop K., Shen H., Meusel M., Rousu J., Böcker S. (2015). Searching molecular structure databases with tandem mass spectra using CSI: FingerID. Proc. Natl. Acad. Sci. USA.

[52] Karonen M., Pihlava J.M. (2022). Identification of Oxindoleacetic Acid Conjugates in Quinoa (*Chenopodium quinoa* Willd.) Seeds by High-Resolution UHPLC-MS/MS. Molecules.

[53] Dührkop K., Fleischauer M., Ludwig M., Aksenov A.A., Melnik A.V., Meusel M., Dorrestein P.C., Rousu J. (2019). SIRIUS SB. 4: A rapid tool for turning tandem mass spectra into metabolite structure information..

[54] Szkudelski T. (2001). The mechanism of alloxan and streptozotocin action in B cells of the rat pancreas. Physiol. Res..

[55] Heikkilä E., Hermant A., Thevenet J., Bermont F., Kulkarni S.S., Ratajczak J., Santo-Domingo J., Dioum E.H., Canto C., Barron D. (2019). The plant product quinic acid activates Ca^2+^-dependent mitochondrial function and promotes insulin secretion from pancreatic beta cells. Br. J. Pharmacol..

[56] Gupta R.C., Chang D., Nammi S., Bensoussan A., Bilinski K., Roufogalis B.D. (2017). Interactions between antidiabetic drugs and herbs: An overview of mechanisms of action and clinical implications. Diabetol. Metab. Syndr..

[57] Singh B., Kumar A., Singh H., Kaur S., Arora S., Singh B. (2022). Protective effect of vanillic acid against diabetes and diabetic nephropathy by attenuating oxidative stress and upregulation of NF-κB, TNF-α and COX-2 proteins in rats. Phytother. Res..

[58] Lakshmi B.S., Sujatha S., Anand S., Sangeetha K.N., Narayanan R.B., Katiyar C., Kanaujia A., Duggar R., Singh Y., Srinivas K. (2009). Cinnamic acid, from the bark of *Cinnamomum cassia*, regulates glucose transport via activation of GLUT4 on L6 myotubes in a phosphatidylinositol 3-kinase-independent manner. J. Diabetes.

[59] Kim J.S., Kwon C.S., Son K.H. (2000). Inhibition of alpha-glucosidase and amylase by luteolin, a flavonoid. Biosci. Biotechnol. Biochem..

[60] Wang S., Li Y., Huang D., Chen S., Xia Y., Zhu S. (2022). The inhibitory mechanism of chlorogenic acid and its acylated derivatives on α-amylase and α-glucosidase. Food Chem..

[61] Oboh G., Ademosun A.O., Ayeni P.O., Omojokun O.S., Bello F. (2015). Comparative effect of quercetin and rutin on α-amylase, α-glucosidase, and some pro-oxidant-induced lipid peroxidation in rat pancreas. Comp. Clin. Pathol..

[62] El-Bassossy T.A., Ahmed F.A. (2024). In vitro anti-diabetic effect and molecular docking study of *Phlomis aurea* components as diabetic enzymes inhibitor. Egypt. J. Chem..

[63] Dubey K., Dubey R., Gupta R., Gupta A. (2021). Exploration of diosmin to control diabetes and its complications-an in vitro and in silico approach. Curr. Comput. -Aided Drug Des..

[64] Chen Y., Geng S., Liu B. (2020). Three common caffeoylquinic acids as potential hypoglycemic nutraceuticals: Evaluation of α-glucosidase inhibitory activity and glucose consumption in HepG2 cells. J. Food Biochem..

[65] Lodhi S., Kori M.L. (2021). Structure–activity relationship and therapeutic benefits of flavonoids in the management of diabetes and associated disorders. Pharm. Chem. J..

[66] Dinda B., Dinda M., Roy A., Dinda S. (2020). Dietary plant flavonoids in prevention of obesity and diabetes. Adv. Protein Chem. Struct. Biol..

[67] Brás N.F., Neves R.P., Lopes F.A., Correia M.A., Palma A.S., Sousa S.F., Ramos M.J. (2021). Combined in silico and in vitro studies to identify novel antidiabetic flavonoids targeting glycogen phosphorylase. Bioorganic Chem..

[68] Ghorbani A. (2017). Mechanisms of antidiabetic effects of flavonoid rutin. Biomed. Pharmacother..

[69] Díaz-de-Cerio E., Girón F., Pérez-Garrido A., Pereira A.S., Gabaldón-Hernández J.A., Verardo V., Segura Carretero A., Pérez-Sánchez H. (2023). Fishing the targets of bioactive compounds from *Psidium guajava* L. leaves in the context of diabetes. Int. J. Mol. Sci..

[70] Ali M., Hassan M., Ansari S.A., Alkahtani H.M., Al-Rasheed L.S., Ansari S.A. (2024). Quercetin and Kaempferol as Multi-Targeting Antidiabetic Agents against Mouse Model of Chemically Induced Type 2 Diabetes. Pharmaceuticals.

[71] Proença C., Ribeiro D., Freitas M., Carvalho F., Fernandes E. (2022). A comprehensive review on the antidiabetic activity of flavonoids targeting PTP1B and DPP-4: A structure-activity relationship analysis. Crit. Rev. Food Sci. Nutr..

[72] Lee L.C., Hou Y.C., Hsieh Y.Y., Chen Y.H., Shen Y.C., Lee I.J., Shih M.C., Hou W.C., Liu H.K. (2021). Dietary supplementation of rutin and rutin-rich buckwheat elevates endogenous glucagon-like peptide 1 levels to facilitate glycemic control in type 2 diabetic mice. J. Funct. Foods.

[73] Gaballah H.H., Zakaria S.S., Mwafy S.E., Tahoon N.M., Ebeid A.M. (2017). Mechanistic insights into the effects of quercetin and/or GLP-1 analogue liraglutide on high-fat diet/streptozotocin-induced type 2 diabetes in rats. Biomed. Pharmacother..

[74] Sharma N., Soni R., Sharma M., Chatterjee S., Parihar N., Mukarram M., Kale R., Sayyed A.A., Behera S.K., Khairnar A. (2022). Chlorogenic acid: A polyphenol from coffee rendered neuroprotection against rotenone-induced Parkinson’s disease by GLP-1 secretion. Mol. Neurobiol..

[75] Kasprzak A. (2021). Insulin-like growth factor 1 (IGF-1) signaling in glucose metabolism in colorectal cancer. Int. J. Mol. Sci..

[76] Hajiaghaalipour F., Khalilpourfarshbafi M., Arya A. (2015). Modulation of glucose transporter protein by dietary flavonoids in type 2 diabetes mellitus. Int. J. Biol. Sci..

[77] Mehta V., Malairaman U. (2016). Flavonoids: Prospective strategy for the management of diabetes and its associated complications. Handbook of Research on Advancing Health Education through Technology.

[78] Yu J., Hu Y., Sheng M., Gao M., Guo W., Zhang Z., Wang D., Wu X., Li J., Chen Y. (2023). Selective PPARγ modulator diosmin improves insulin sensitivity and promotes browning of white fat. J. Biol. Chem..

[79] Cai Y., Fan C., Yan J., Tian N., Ma X. (2012). Effects of rutin on the expression of PPARγ in skeletal muscles of db/db mice. Planta Medica.

[80] Lokhande K.B., Ballav S., Thosar N., Swamy K.V., Basu S. (2020). Exploring conformational changes of PPAR-Ɣ complexed with novel kaempferol, quercetin, and resveratrol derivatives to understand binding mode assessment: A small-molecule checkmate to cancer therapy. J. Mol. Model..

[81] Chiu M.L., Chiou J.S., Chen C.J., Liang W.M., Tsai F.J., Wu Y.C., Lin T.H., Liao C.C., Huang S.M., Chou C.H. (2022). Effect of Chinese herbal medicine therapy on risks of overall, diabetes-related, and cardiovascular diseases-related mortalities in Taiwanese patients with hereditary hemolytic anemias. Front. Pharmacol..

[82] Qaid M.M., Abdelrahman M.M. (2016). Role of insulin and other related hormones in energy metabolism—A review. Cogent Food Agric..

[83] Ali N., Diamond D.M., Rice S.M. (2023). Cardiovascular disease and its association with insulin resistance and cholesterol. Ketogenic.

[84] Sobczak I.S.A., Blindauer A.C., Stewart J.A. (2019). Changes in plasma free fatty acids associated with type-2 diabetes. Nutrients.

[85] Kim M.J., Sim D.Y., Lee H.M., Lee H.J., Kim S.H. (2019). Hypolipogenic effect of shikimic acid via inhibition of MID1IP1 and phosphorylation of AMPK/ACC. Int. J. Mol. Sci..

[86] Kang H., Koppula S. (2014). *Houttuynia cordata* attenuates lipid accumulation via activation of AMP-activated protein kinase signaling pathway in HepG2 cells. Am. J. Chin. Med..

[87] Alam M.M., Meerza D., Naseem I. (2014). Protective effect of quercetin on hyperglycemia, oxidative stress and DNA damage in alloxan induced type 2 diabetic mice. Life Sci..

[88] Arya A., Al-Obaidi M.M., Shahid N., Noordin M.I., Looi C.Y., Wong W.F., Khaing S.L., Mustafa M.R. (2014). Synergistic effect of quercetin and quinic acid by alleviating structural degeneration in the liver, kidney and pancreas tissues of STZ-induced diabetic rats: A mechanistic study. Food Chem. Toxicol..

[89] McLeish M.J., Kenyon G.L. (2005). Relating Structure to Mechanism in Creatine Kinase. Crit. Rev. Biochem. Mol. Biol..

[90] Loubani M., Fowler A., Standen N.B., Galiñanes M. (2005). The effect of gliclazide and glibenclamide on preconditioning of the human myocardium. Eur. J. Pharmacol..

[91] Bati K., Baeti P.B., Gaobotse G., Kwape T.E. (2024). Leaf extracts of *Euclea natalensis* A.D.C ameliorate biochemical abnormalities in high-fat-low streptozotocin-induced diabetic rats through modulation of the AMPK-GLUT4 pathway. Egypt. J. Basic Appl. Sci..

[92] Galic S., Loh K., Murray-Segal L., Steinberg G.R., Andrews Z.B., Kemp B.E. (2018). AMPK signaling to acetyl-CoA carboxylase is required for fasting- and cold-induced appetite but not thermogenesis. Elife.

[93] Zhang T., Xu L., Guo X., Tao H., Liu Y., Liu X., Zhang Y., Meng X. (2023). The potential of herbal drugs to treat heart failure: The roles of Sirt1/AMPK. J. Pharm. Anal..

[94] Wang J.H., Bose S., Lim S.K., Ansari A., Chin Y.W., Choi H.S., Kim H. (2017). *Houttuynia cordata* facilitates metformin on ameliorating insulin resistance associated with gut microbiota alteration in OLETF rats. Genes.

